# Precision screening facilitates clinical classification of BRCA2-PALB2 binding variants with benign and pathogenic functional effects

**DOI:** 10.1172/JCI181879

**Published:** 2025-04-15

**Authors:** Muthiah Bose, Manika Indrajit Singh, Morten Frödin, Bent Ejlertsen, Claus S. Sørensen, Maria Rossing

**Affiliations:** 1 Center for Genomic Medicine, Rigshospitalet, Copenhagen University Hospital, Copenhagen, Denmark.; 2 Biotech Research and Innovation Center, Faculty of Health and Medical Sciences, University of Copenhagen, Copenhagen, Denmark.; 3 Department of Clinical Oncology, Rigshospitalet, Copenhagen University Hospital, Copenhagen, Denmark.; 4 Department of Clinical Medicine, Faculty of Health and Medical Sciences, University of Copenhagen, Copenhagen, Denmark.

**Keywords:** Genetics, Oncology, Breast cancer, DNA repair, Genetic diseases

## Abstract

**BACKGROUND:**

Decoding the clinical impact of genetic variants is particularly important for precision medicine in cancer. Genetic screening of mainly patients with breast and ovarian cancer has identified numerous *BRCA1*/*BRCA2* variants of uncertain significance (VUS) that remain unclassified owing to a lack of pedigrees and functional data.

**METHODS:**

Here, we used CRISPR-Select — a technology that exploits unique inbuilt controls at the endogenous locus — to assess 54 rare ClinVar VUS located in the PALB2-binding domain of *BRCA2*. Variant deleteriousness was examined in the absence and presence of PARPi, cisplatin, or mitomycin C.

**RESULTS:**

Marked functional deficiency was observed for variants in the exon 2 donor splice region (A22 = c.66A>C, A22 = c.66A>G, A22 = c.66A>T, and D23H) and Trp31 aa (W31G, W31L, and W31C), both critical for *BRCA2* function. Moreover, T10K and G25R resulted in an intermediate phenotype, suggesting these variants are hypomorphic in nature. Combining our functional results with the latest ClinGen *BRCA1/2* Variant Curation Expert Panel recommendations, we classified 49 of the 54 VUS as either likely benign (*n* = 45) or likely pathogenic (*n* = 4).

**CONCLUSION:**

Therefore, CRISPR-Select is an important tool for efficient variant clinical classification. Application of this technology in the future will ultimately improve patient care.

**FUNDING:**

Danish Cancer Society, Novo Nordisk Foundation, Sygeforsikring Danmark, Børnecancerfonden, Neye-Fonden, Roche, Novartis, Pfizer, AstraZeneca, MSD, and Daiichi Sankyo Europe GmbH.

## Abstract 2

In this study, genetic varaints with unknown function has been tested in laboratory cellular models, enabling the identification of its cancer relevance <div> </div>

## Introduction

Inherited pathogenic variants in *BRCA2* confer an average cumulative lifetime risk of developing breast and ovarian cancer of about 69% and 17%, respectively (1). In addition, a significantly increased risk of developing prostate, pancreatic, and stomach cancer is observed among *BRCA2* pathogenic variant carriers (2, 3). *BRCA2* genetic testing to identify patients with pathogenic variants is now a priority, as affected patients can benefit from effective clinical management such as intensified screening programs, prophylactic surgery, presymptomatic genetic testing of family members, and targeted treatment using poly-ADP-ribose polymerase inhibitors (PARPi) (4).

Wider use of genetic testing has resulted in the identification of large numbers of individual *BRCA2* variants, which are routinely classified using the 5-tier variant classification system (5–7). Germline testing is expected to increase owing to a new guideline issued by the American Society of Clinical Oncology in partnership with the Society of Surgical Oncology, which recommends screening of all newly diagnosed patients with breast cancer ≤65 years regardless of family history (8). In addition to in silico analysis, the current variant classification system relies heavily on genetic and clinical data, such as segregation analysis, family history, and tumor characteristics, for accurate classification of variants (9–11). However, because many of the observed variants are rare, the genetic and clinical data are insufficient for determining a decisive classification, and these variants are termed variants of uncertain significance (VUS) (11). As the vast majority of variants reported in *BRCA2* (3,341 in total) are VUS (6), this presents a major challenge for the clinical management of carriers, both healthy individuals and patients with cancer. Therefore, accurate classification would have a profound clinical impact for *BRCA2* VUS carriers.

In order to accurately classify VUS, major efforts have been directed toward establishing comprehensive consortia for efficient data sharing and collaborations, such as Evidence-based Network for the Interpretation of Germline Mutant Alleles (ENIGMA), which is considered the gold standard among consortia (11, 12). Moreover, several functional assays have recently been developed that could support an accurate classification of VUS following the guidelines of the FDA-approved Clinical Genome Resource (ClinGen; https://clinicalgenome.org/) *BRCA1/2* Variant Curation Expert Panel (VCEP) (4, 13–18). Results from well-established functional assays can be applied at “strong” evidence levels (PS3/BS3) as per the American College of Medical Genetics and Genomics (ACMG) and the Association for Molecular Pathology (AMP) variant classification system (19). For rare variants, classification using the ClinGen *BRCA1/2* VCEP model relies heavily on well-calibrated functional assays, due to limited information from other types of evidence (18).

According to ClinGen *BRCA1/2* VCEP, only the PALB2-binding domain (PBD, aa 10–40) and the DNA-binding domain (DBD, aa 2,481–3,186) are deemed to be clinically important functional domains in BRCA2 (18). To date, a significant number of studies have focused on classifying VUS identified in the *BRCA2*-DBD, largely due to its functional importance and cancer relevance (4, 14, 20–22). To the best of our knowledge, there are currently no studies that have systematically mapped the functional effects of multiple VUS observed in the entire PBD of *BRCA2*. Here, using the recently developed quantitative CRISPR-Select approach (23), we have successfully mapped the functional effects of 54 rare ClinVar VUS (https://www.ncbi.nlm.nih.gov/clinvar/) distributed throughout *BRCA2*-PBD. Our analysis has confirmed that both the exon 2 donor splice region and Trp31 aa in the PBD are essential for *BRCA2* function. Moreover, our results highlight the efficacy and utility of the CRISPR-Select functional assay in providing clinical classification of VUS identified in a clinical setting.

## Results

**Figure 1:**
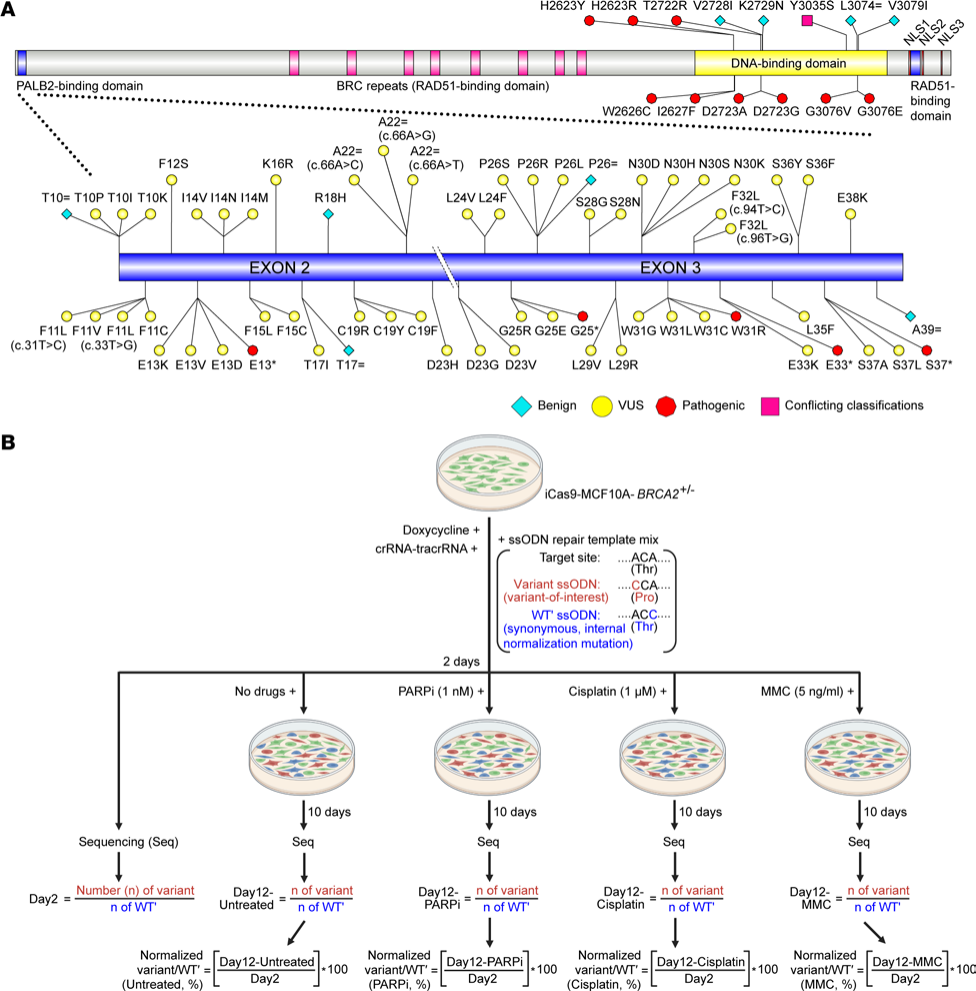
Variants assessed in this study using CRISPR-Select. (**A**) An overview of 78 variants (benign/likely benign, 9; VUS, 54; likely pathogenic/pathogenic, 14; conflicting classifications, 1) selected from ClinVar for functional assessment using CRISPR-Select (58). Experimental control variants were selected from both the PALB2-binding domain and the DNA-binding domain. W2626C was utilized as both a pathogenic control and a low-penetrance/risk allele, while Y3035S was considered exclusively as a low-penetrance/risk allele. (**B**) Schematic representation of the CRISPR-Select assay employed in this study for variant classification. The counts of variant- and WT′-containing reads are used to evaluate the deleteriousness of genetic variants by calculating the variant/WT′ ratio for Day2, Day12-Untreated, Day12-PARPi, Day12-Cisplatin, and Day12-MMC samples. These ratios are then normalized to the Day2-variant/WT′ ratio which is set at 100%. These ratios are then normalized to the day 2–variant/WT′ ratio, which was set at 100%. The normalized variant/WT′ ratios were then averaged across biological replicates and are reported as mean ± SD in Figures 2 and 3.

Of the clinically important domains in *BRCA2*, VUS located in the DBD have been relatively well characterized using a plethora of different functional assays. However, until now, VUS located in the PBD have not been systematically assessed. Accordingly, after consulting ClinVar, we shortlisted 54 rare VUS distributed between *BRCA2* exon 2 (25 VUS) and exon 3 (29 VUS) (Figure 1A and Supplemental Table 1; supplemental material available online with this article; https://doi.org/10.1172/JCI181879DS1). Additionally, to estimate the odds of pathogenicity (OddsPath) for CRISPR-Select and establish assay threshold, we selected 9 previously classified benign/likely benign and 14 previously classified likely pathogenic/pathogenic variants (Figure 1A and Supplemental Table 1). Due to limited availability of suitable variants within the PBD alone, experimental control variants were selected from both PBD and DBD (Figure 1A).

Moreover, to investigate the functional impact of known low-penetrance/risk allele variants, Y3035S (classified with conflicting interpretations in ClinVar) was included alongside W2626C (pathogenic in ClinVar) (Figure 1A and Supplemental Table 1). The inclusion of additional low-penetrance/risk allele variants was limited, as only W2626C and Y3035S have been evaluated for disease penetrance or association in large clinical cohorts (24–26). These 78 variants (benign/likely benign, 9; VUS, 54; likely pathogenic/pathogenic, 14; conflicting classifications, 1) were further functionally assessed utilizing CRISPR-Select (Figure 1B and Supplemental Table 1).

We used CRISPR-Select owing to its unique feature of inbuilt internal controls that allow precise simultaneous functional testing of VUS and synonymous (WT′) variants in the same cell culture dish, a key control in functional assays (27). Thus, cells were transfected with a mixture of *BRCA2*-targeting single-stranded oligodeoxynucleotides (ssODNs), and the ratios between variants were determined using NGS during the course of the experiment (Figure 1B) (23). Variant deleteriousness was examined in the absence and presence of PARPi (1 nM), cisplatin (1 μM), or mitomycin C (MMC) (5 ng/mL) (Figure 1B and Supplemental Figure 1).

Receiver operating characteristic (ROC) curve analysis was performed on normalized variant/WT′ ratios for the included 9 benign/likely benign variants and 14 likely pathogenic/pathogenic variants. Based on this analysis, the following thresholds were established to classify variants as neutral (variant/WT′ ratio, >50%), intermediate (variant/WT′ ratio, 25%–50%), or deleterious (variant/WT′ ratio, ≤25%) (Supplemental Table 2) (28). Similar thresholds were applied evaluating variant effects in both the absence and presence of PARPi, cisplatin, or MMC. Using these experimental controls, sensitivity and specificity of the assay in the presence or absence of drugs were estimated at 100%, with 95% CIs of 78.5%–100% for sensitivity and 70.1%–100% for specificity. Of note, Y3035S was excluded from the sensitivity, specificity, and threshold estimation of CRISPR-Select, owing to its conflicting classifications in ClinVar.

**Figure 2:**
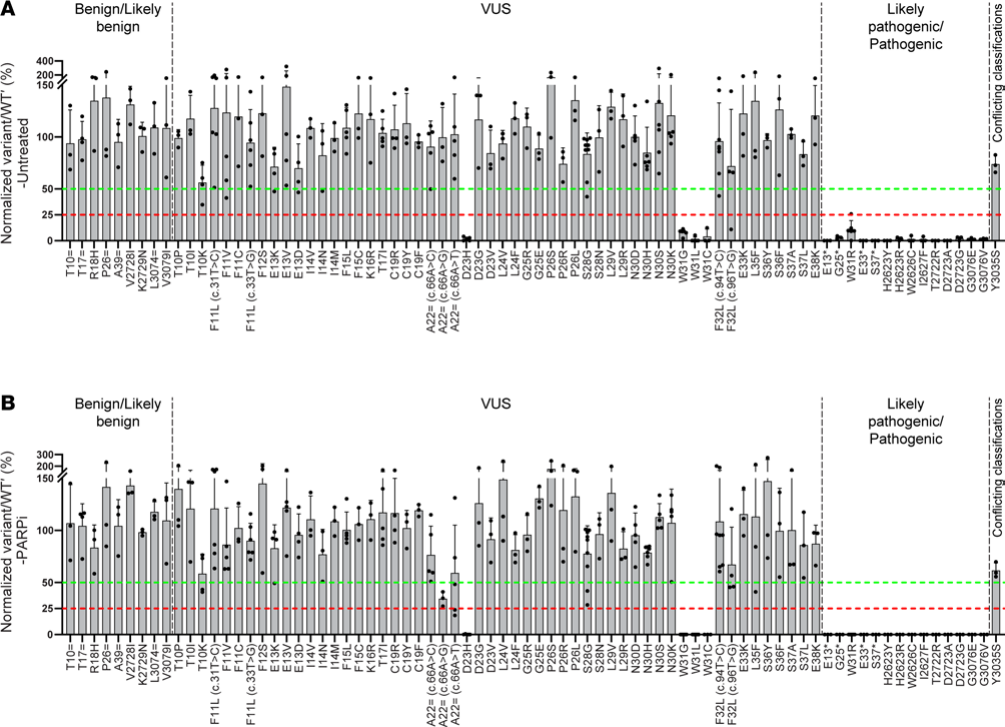
Normalized variant/WT′ ratios under normal cell-culturing conditions and PARPi treatment. The normalized values for each variant were consolidated into a single plot, as shown in **A** (Untreated) and **B** (PARPi). Data are shown as the mean ± SD of a minimum of 3 independent biological replicates. Assay thresholds were established using ROC curve analysis to classify variants as neutral (variant/WT′ ratio, >50%), intermediate (variant/WT′ ratio, 25%–50%), or deleterious (variant/WT′ ratio, ≤25%). The red dotted lines represent the threshold for deleterious variants, while the green dotted lines represent the threshold for neutral variants.

**Figure 3:**
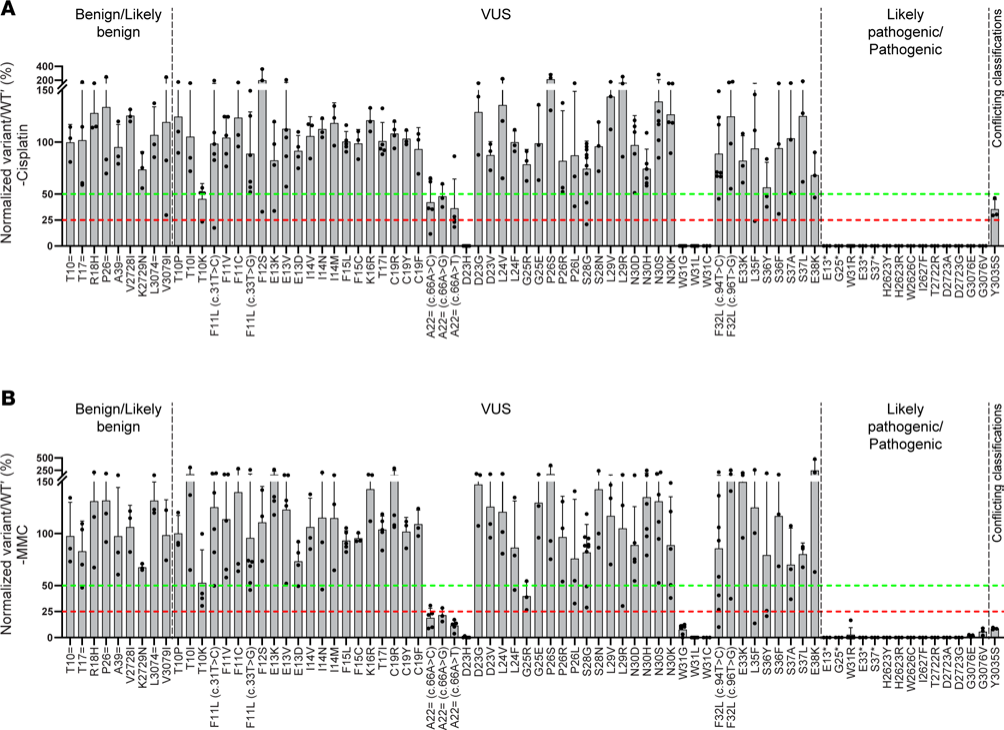
Normalized variant/WT′ ratios under cisplatin and MMC treatment. The normalized values for each variant were consolidated into a single plot, as shown in **A** (cisplatin) and **B** (MMC). Data are shown as the mean ± SD of a minimum of 3 independent biological replicates. Assay thresholds were established using ROC curve analysis to classify variants as neutral (variant/WT′ ratio, >50%), intermediate (variant/WT′ ratio, 25%–50%), or deleterious (variant/WT′ ratio, ≤25%). The red dotted lines represent the threshold for deleterious variants, while the green dotted lines represent the threshold for neutral variants.

Under normal cell-culturing conditions (untreated), together with the (likely) pathogenic variant controls, VUS located in the exon 2 donor splice region (D23H) and all Trp31 alterations (W31G, W31L, and W31C) displayed deleteriousness (Figure 2A and Supplemental Table 2). The remaining VUS, together with the (likely) benign variant controls, exhibited a neutral phenotype under normal growth conditions (Figure 2A). During PARPi (Figure 2B) and cisplatin (Figure 3A) treatment, no additional VUS displayed deleteriousness. In line with untreated conditions, complete loss of viability was observed for D23H, W31G, W31L, and W31C (Figure 2B, Figure 3A, and Supplemental Table 2). Furthermore, VUS such as T10K, A22 = c.66A>C, A22 = c.66A>G, and A22 = c.66A>T displayed an intermediate effect upon PARPi and cisplatin treatment (Figure 2B and Figure 3A).

In addition to D23H and Trp31 alterations (W31G, W31L, and W31C), the MMC sensitivity assay (unlike PARPi/cisplatin) was able to identify 3 additional deleterious variants (A22 = c.66A>C, A22 = c.66A>G, and A22 = c.66A>T) located in the exon 2 donor splice region (Figure 3B and Supplemental Table 2). Moreover, only G25R displayed an intermediate effect upon MMC treatment, which has previously been demonstrated to be hypomorphic in nature (29) (Figure 3B). Regarding low-penetrance/risk allele variants, W2626C exhibited deleterious effects both in the absence and presence of PARPi, cisplatin, or MMC, whereas Y3035S showed deleterious effects only upon MMC treatment (Figures 2 and 3). Thus, inclusion of an MMC-based sensitivity assay has yielded additional information regarding variant effects, thereby strongly indicating its inclusion would be beneficial in future VUS assessments.

**Figure 4:**
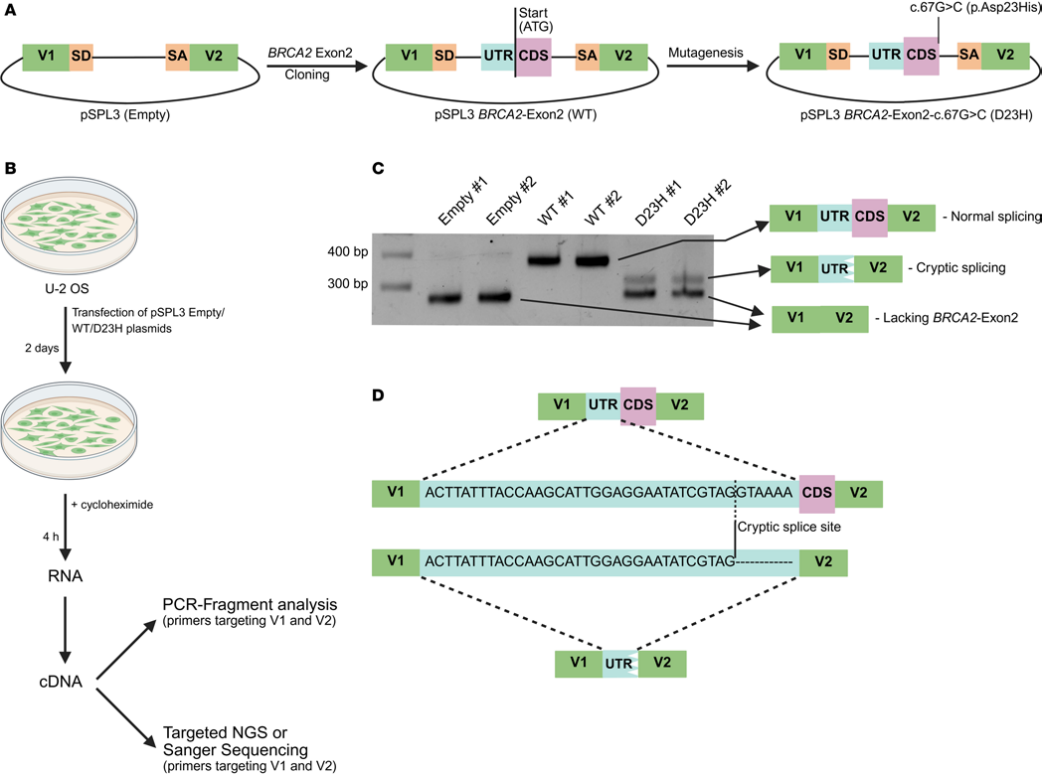
Minigene splicing assay illustrating aberrant splicing of *BRCA2* c.67G>C (D23H) variant. (**A**) WT *BRCA2* exon 2 and flanking intronic sequences were PCR amplified and cloned into pSPL3 vector. pSPL3 *BRCA2*-Exon2-c.67G>C (D23H) plasmids were generated using site-directed mutagenesis. V1, vector exon 1; SD, splice-donor sequence; SA, splice-acceptor sequence; V2, vector exon 2; UTR, untranslated region; CDS, coding sequence. (**B**) pSPL3-Empty, pSPL3-WT, and pSPL3-D23H plasmids were transfected into U-2-OS cells and treated with 300 μg/mL cycloheximide to block nonsense-mediated mRNA decay before RNA purification. cDNA was sequenced and analyzed using pSPL3 specific primers targeting V1 and V2. (**C**) PCR fragment analysis indicating the presence of 2 independent aberrant splicing patterns of the *BRCA2* c.67G>C (D23H) variant. Skipping of *BRCA2* exon 2 (D23H #1 and #2, bottom band) was the predominant aberrant splicing pattern, whereas use of cryptic splice site (D23H #1 and #2, top band) that results in incorporation of truncated UTR segment was also observed. (**D**) Targeted NGS of *BRCA2*-Exon2-c.67G>C (D23H) cDNA revealed the use of a noncanonical splice site located upstream of the CDS, resulting in an aberrantly spliced product lacking part of UTR and entire CDS.

Among these deleterious variants, D23H is located in the exon 2 donor splice region adjacent to the splice donor dinucleotide site. The deleteriousness observed with D23H could be either due to splicing defects or aa change impacting protein binding or stability. To uncover splicing impacts associated with D23H, a fragment including *BRCA2* exon 2 along with flanking intronic sequences was cloned into the minigene vector pSPL3 containing exons from HIV-tat and rabbit β-globin genes under the control of SV40 promoter (Figure 4A) (30). pSPL3 *BRCA2*-Exon2-c.67G>C (D23H) plasmid was generated through site-directed mutagenesis along with pSPL3-Empty and pSPL3-WT transfected into U-2-OS cells (Figure 4, A and B). PCR fragment analysis indicated the presence of two independent aberrant splicing patterns of D23H variant (Figure 4C). Targeted next-generation sequencing (NGS) or Sanger sequencing in these aberrantly spliced products revealed skipping of *BRCA2* exon 2 (D23H #1 and #2, bottom band, Figure 4C) as predominant outcome, whereas activation of cryptic splice site (GT) located upstream of coding sequences (CDS) resulting in a truncated product that lacks the entire CDS and part of UTR was also observed (Figure 4D). These results indicate that the deleteriousness observed with D23H is primarily due to aberrant splicing.

**Figure 5:**
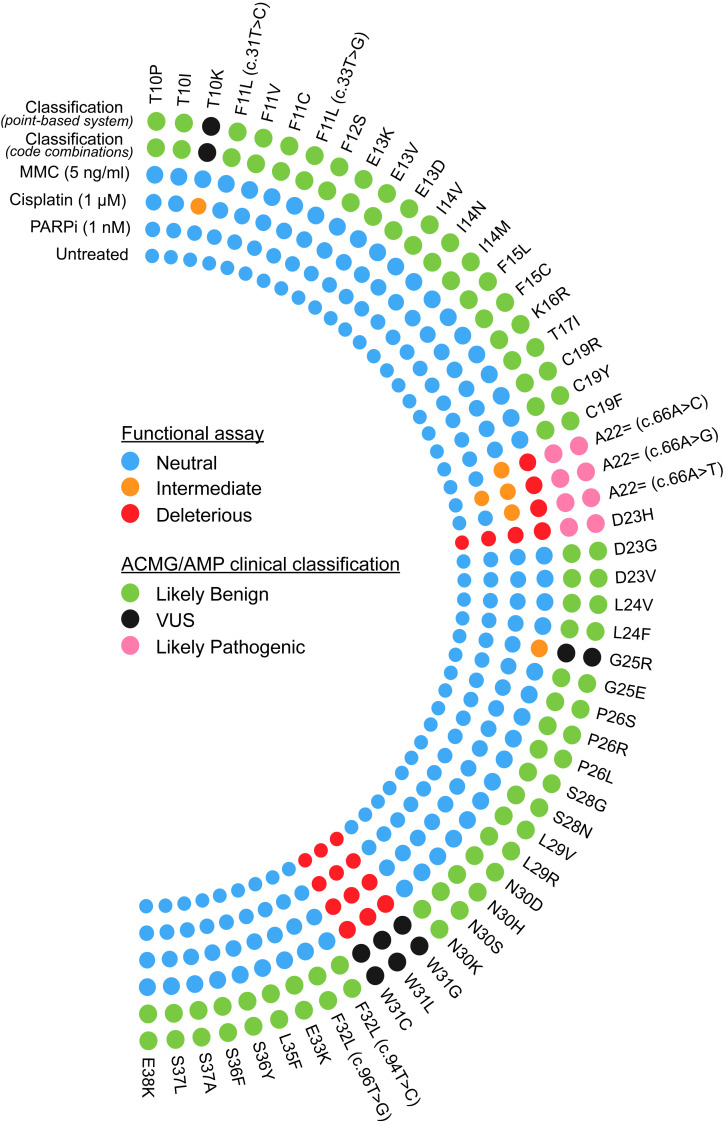
Circos plot depicting the functional consequence and clinical classification of each VUS assessed in this study. Variants were defined as neutral (variant/WT′ ratio, >50%), intermediate (variant/WT′ ratio, 25%–50%), or deleterious (variant/WT′ ratio, ≤25%) based on their functional effects. VUS were clinically classified utilizing the latest ClinGen *BRCA1/2* VCEP recommendations under both qualitative ACMG/AMP code combinations and a point-based system.

Using the functional assay results, our next objective was to clinically classify these VUS (*n* = 54) by applying the latest ClinGen *BRCA1/2* VCEP recommendations (Figure 5 and Supplemental Table 3) (18). ACMG/AMP clinical codes such as PS3/BS3 (functional assay), PM2/BA1/BS1 (population data), PP3/BP4 (computational evidence), PS1(Splicing) (predicted splicing event), and PVS1_Strong (RNA) (splicing assay data) were employed, and a detailed description regarding their use is provided in Methods. Specifically, the code PS3-Moderate was assigned to variants showing deleterious effects in either untreated or drug sensitivity assays, while the code PS3-Indeterminate was applied to variants displaying an intermediate phenotype without deleteriousness in either assay. The code BS3-Moderate was assigned to all neutral variants in both untreated and drug sensitivity assays (Supplemental Table 3). Finally, using both qualitative ACMG/AMP code combinations and a point-based system, we were able to classify 49 of the 54 VUS as likely benign (*n* = 45) and likely pathogenic (*n* = 4) (Figure 5 and Supplemental Table 3).

ClinGen *BRCA1/2* VCEP recommends using the BayesDel tool to predict the deleterious nature of VUS observed in *BRCA1/2* (18). Specifically for *BRCA2*, BP4 (BayesDel_noAF ≤0.18) and PP3 (BayesDel_noAF ≥0.30) could be applied based on the prediction strength. BayesDel provides a deleteriousness metascore by combining pathogenicity scores from multiple tools, including PolyPhen-2, SIFT, FATHMM, LRT, MutationTaster, MutationAssessor, phyloP score, GERP++, and SiPhy (31, 32). While applying PP3/BP4, we noticed that none of our VUS (*n* = 54) had a BayesDel_noAF score (≥0.30) in favor of pathogenicity (Supplemental Table 3), despite our assay indicating that the exon 2 donor splice region and Trp31 aa are essential for BRCA2.

Different alterations in *BRCA2*-PBD with a BayesDel score in favor of pathogenicity (PP3) may exist, which could be assessed to determine the concordance between BayesDel prediction and our assay. To identify such variants, we analyzed the BayesDel score for all possible substitution variants in *BRCA2*-PBD and found that none of the possible missense alterations had a BayesDel score in favor of pathogenicity (Supplemental Table 4). Only nonsense variants had a BayesDel score ≥0.30, except *BRCA2* c.61A>T p.(Lys21Ter) with a BayesDel_noAF score of 0.294 (Supplemental Table 4).

These results indicate that under current ClinGen *BRCA1/2* VCEP recommendations, PP3 could not be applied for any missense VUS observed in *BRCA2*-PBD (Supplemental Tables 3 and 4). The absence of PP3 affects our potential to classify VUS as likely pathogenic/pathogenic, since only PP3 and PM2 are readily available for these types of rare VUS. Most importantly, under the soon-to-be implemented ACMG/AMP point-based system, all missense variants in *BRCA2*-PBD would be awarded negative points (–1) due to BP4 assignment (Supplemental Table 3). Despite all Trp31 alterations (W31R [control], W31G, W31L, and W31C) in our assay displaying a severe damaging phenotype, we were unable to classify these variants owing to BP4 (instead of PP3) and PS3 at moderate evidence strength (Figure 5 and Supplemental Table 3).

Next, we investigated the concordance between Align-GVGD (A-GVGD) prediction and our assay. Unlike metapredictors (BayesDel and NVM_predict), A-GVGD uses biophysical characteristics of aa and protein multiple sequence alignments to predict the variant effect (33, 34). In agreement with our assay, deleterious variants such as W31R (C65), W31G (C65), W31L (C55), and W31C (C65) were also predicted to be damaging by the A-GVGD algorithm, whereas G25R (C65) displayed an intermediate phenotype upon MMC treatment (Figures 2, 3, and 5 and Supplemental Table 3). However, in contrast, neutral variants in our assay, such as F11C (C55), F15C (C65), and G25E (C65), were estimated to be deleterious by the A-GVGD algorithm (Figures 2, 3, and 5 and Supplemental Table 3). Therefore, we observed better agreement between our assay and A-GVGD prediction than with the BayesDel tool. Nevertheless, A-GVGD overestimates the variant effect (at least for F11C, F15C, and G25E) owing to changes in the properties of aa side chains.

In conclusion, we functionally assessed 54 rare VUS using CRISPR-Select and confirmed that both the exon 2 donor splice region and the Trp31 aa in the PBD are critical for BRCA2 function. Moreover, using the latest ClinGen *BRCA1/2* VCEP recommendations, we were able to classify 49 of the 54 VUS as likely benign (*n* = 45) and likely pathogenic (*n* = 4). Thus, the newly developed CRISPR-Select assay has proven highly suitable for functional variant analysis. Finally, we observed that PP3 could not be applied for any missense VUS located in *BRCA2*-PBD, which will impact future variant classification using the ACMG/AMP point-based scoring approach.

## Discussion

High-caliber functional assays are recognized as an important support for the clinical classification of variants in genetic diseases. The recently developed quantitative CRISPR-Select assay is extremely versatile, with a rapid turnaround. Importantly, it can be readily implemented by various diagnostic and research laboratories as it only requires the delivery of standard CRISPR-Cas9 reagents to cells together with a simplified analysis pipeline for VUS classification (Figure 1B). Using CRISPR-Select, different types of genetic alterations such as splice sites, missense mutations, and indels can be readily assessed in proper genomic/cellular contexts (23). Potential experimental confounders such as CRISPR off-target effects, varying transfection efficiency/toxicity, cell density, edge effects, etc., are normalized in the assay due to the inclusion of a synonymous internal normalization mutation (WT′) (23) (Figure 1B). Moreover, CRISPR-Select encompasses an additional internal control for null effect, and such frameshift indels due to CRISPR-Cas9 activity can be tracked under a range of conditions. The present analysis has demonstrated a strong negative selection against cells with frameshift indels in the same cell culture dish, where the benign/likely benign and neutral variants were not selected against (Supplemental Figure 2), thus providing further confidence in the use of this assay for variant classification.

In this study, using an appropriate nontumorigenic human mammary epithelial cell line (MCF10A), we have successfully (re)classified 49 of 54 rare VUS as likely benign (*n* = 45) and likely pathogenic (*n* = 4) (Figure 5 and Supplemental Table 3). Adding additional lines of ACMG/AMP evidence would enhance our ability to effectively classify additional VUS, but as mentioned above, since these VUS are rare, this was not possible for the majority of ACMG/AMP evidence criteria. In particular, PP1/BS4 (disease cosegregation) and PP4/BP5 (multifactorial likelihood analysis) evidence could not be systematically applied in our study, as previously published studies only had likelihood ratios (LRs) for a very few *BRCA2*-PBD VUS (35–38).

Similarly, PS4 (case-control LR) and PM3/BS2 (co-occurrence in Fanconi anemia) evidence could not be applied, as the variants assessed here had not previously been reported to be associated with breast cancer and Fanconi Anemia (39, 40). However, the recent BRIDGES study has reported the presence of a few undisclosed *BRCA2*-PBD variants among patients with breast cancer, which could subsequently be explored to identify the PS4 strength of these variants (41, 42). It should be noted that, presently, most of the reported clinically relevant breast cancer and Fanconi anemia genetic variants are located in *BRCA2*-DBD and not in PBD.

Our assay included 9 benign/likely benign and 14 likely pathogenic/pathogenic controls. Thus, the OddsPath was 9 for pathogenic and 0.071 for benign variants, enabling us to use PS3/BS3 at moderate evidence strength. By assessing more control variants from the DBD, the evidence strength of PS3/BS3 for CRISPR-Select will increase accordingly. Additionally, variants with “conflicting classifications” could subsequently be explored, as only VUS in *BRCA2*-PBD were addressed in this study. In the future, ACMG/AMP evidence criteria such as PP1/BS4, PP4/BP5, PS4, and PM3/BS2 could be applied for *BRCA2*-PBD VUS through data sharing and collaborations with international consortia.

We have highlighted that due to current ClinGen *BRCA1/2* VCEP recommendations, we could not apply PP3 for any missense alteration in *BRCA2*-PBD owing to the BayesDel_noAF score of ≤0.18 (Supplemental Tables 3 and 4). This impacted our ability to classify the VUS, since only PP3 and PM2 are readily available ACMG/AMP evidence criteria for many rare VUS. As detailed in the ClinGen *BRCA1/2* VCEP recommendations, metapredictors such as BayesDel and NVM_predict are superior in comparison to other tools in predicting variant effects in functional assays (18, 31, 34). However, we identified a better correlation between the results of our functional assays and A-GVGD prediction rather than the BayesDel tool (Supplemental Table 3). We believe this discrepancy is due to the composition of the functional assay reference dataset used by the ClinGen *BRCA1/2* VCEP panel to derive LRs for the identification of optimal bioinformatic prediction tools. Specifically, among 238 *BRCA2* reference variants (with known functional impact) used to derive LRs, only 3 (R18H, G25R, and W31C) are in *BRCA2*-PBD, whereas most of the remaining variants are in *BRCA2*-DBD. Thus, we propose that the BayesDel tool should be used to predict the variant effect for genetic alterations located in *BRCA2*-DBD, whereas other prediction tools (with sufficient threshold optimization by considering sensitivity and specificity) should be used for variants located in *BRCA2*-PBD. Since our dataset contained only 6 deleterious variants, we were unable to derive reliable LRs; however, this could be achieved by saturation genome editing in *BRCA2*-PBD.

Our study has confirmed that both the exon 2 donor splice region and Trp31 aa in the PBD are critical for *BRCA2* function. Moreover, alterations such as T10K and G25R resulted in an intermediate phenotype, thus depicting their hypomorphic nature. The deleterious nature of W31 alterations (W31R, W31G, W31L, and W31C) in our assay may be due to either abrogation of PALB2 binding or partial exon 3 skipping, as detailed by Thomassen et al. (43). It should be noted that they reported only minimal (~10%) partial *BRCA2* exon 3 skipping with W31 alterations (43), indicating that the high level of impact observed here was potentially due to abrogated BRCA2-PALB2 binding (Figures 2, 3, and 5).

The exon 2 donor splice region is intriguing; the closest alteration (D23H) to the splice donor ±1,2 dinucleotide position was found to be highly damaging; mutations in 1 codon before D23H (A22 = (c.66A>C), A22 = (c.66A>G), and A22 = (c.66A>T)) were also damaging but not to the same extent as D23H (Figure 2, 3, and 5). In addition to the exon 2 donor splice region and Trp31, other regions/aa may also be critical in the PBD, which could be addressed by performing saturation genome editing. Of note, VUS such as A22 = c.66A>C, A22 = c.66A>G, and A22 = c.66A>T, along with the low-penetrance variant Y3035S (c.9104A>C), exhibit deleterious effects exclusively with MMC treatment and not with PARPi or cisplatin. While this observation is based on only 4 variants, it suggests that MMC sensitizes cells through a distinct mechanistic pathway, thereby strongly suggesting its inclusion in future VUS assessments.

The key limitation of CRISPR-Select is its low throughput regarding the number of tested variants compared with multiplexed assays of variant effects (MAVEs), such as deep mutational scans and massively parallel reporter assays (17, 20–22). Nevertheless, as CRISPR-Select is extremely sensitive and readily available, with a rapid turnaround, it is highly advantageous in the clinical/diagnostic setting. Moreover, CRISPR-Select is particularly applicable to the investigation of VUS not being assessed in MAVE assays, variants showing conflicting evidence between different MAVE assays or a given variant that is being tested under a range of conditions (such as different time points or drugs). Furthermore, unlike MAVE assays, implementation of CRISPR-Select is straightforward as it uses standard CRISPR-Cas9 reagents with a simplified analysis pipeline, thus making it readily available for multiple entities.

Owing to the genomic era, more VUS are being identified in clinical diagnostics, stressing the need for efficient and robust variant classification approaches. Our study is a clear example of the benefits when basic research and clinical guidelines are combined. We present here an action plan on how to execute functional assays together with the application of clinical guidelines to ultimately achieve optimized patient care and management. The CRISPR-Select assay and its associated analysis are straightforward to perform, and the functional readouts readily support the standardized ClinGen guidelines. The overall objective for the future is to be able to provide a clinical classification for each individual patient’s variant and thereby support their personalized medicine program.

## Methods

### Sex as a biological variable

This study did not include any humans and/or animal models.

### Selection of BRCA2 VUS and experimental control variants

In this study, the PBD in BRCA2 was considered between aa 10 and 40 based on the ClinGen *BRCA1/2* VCEP recommendations and Xia et al. (44). In ClinVar, all missense and synonymous VUS located in *BRCA2* aa 10–40 (as of September 6, 2022) were selected for assessment. In total, 54 VUS were shortlisted, distributed between *BRCA2* exon 2 (25 VUS: T10P, T10I, T10K, F11L c.31T>C, F11V, F11C, F11L c.33T>G, F12S, E13K, E13V, E13D, I14V, I14N, I14M, F15L, F15C, K16R, T17I, C19R, C19Y, C19F, A22 = c.66A>C, A22 = c.66A>G, A22 = c.66A>T, D23H) and exon 3 (29 VUS: D23G, D23V, L24V, L24F, G25R, G25E, P26S, P26R, P26L, S28G, S28N, L29V, L29R, N30D, N30H, N30S, N30K, W31G, W31L, W31C, F32L c.94T>C, F32L c.96T>G, E33K, L35F, S36Y, S36F, S37A, S37L, E38K) (Figure 1A and Supplemental Table 1).

Five previously classified benign/likely benign (T10=, T17=, R18H, P26=, and A39=) and 5 previously classified likely pathogenic/pathogenic (E13*, G25*, W31R, E33*, and S37*) variants in PBD were selected from ClinVar to serve as experimental controls (Figure 1A and Supplemental Table 1). Missense variants were prioritized for use as experimental controls. Currently, only *BRCA2* R18H and W31R are classified as benign and likely pathogenic, respectively. To address this limitation, additional missense variants from the DBD were selected from ClinVar, including benign/likely benign variants (V2728I, K2729N, L3074=, and V3079I) and likely pathogenic/pathogenic variants (H2623Y, H2623R, W2626C, I2627F, T2722R, D2723A, D2723G, G3076E, and G3076V) (Figure 1A).

Calibration of specific functional assays using missense control variants from multiple domains remains under deliberation. Two independent surveys conducted at the ENIGMA meeting (45) and the Clinical Application of MAVE Data workshop (46) recommend domain-specific calibration for individual assays. However, as the loss-of-function mechanism in BRCA2 is consistent across clinically relevant domains, we calibrated CRISPR-Select using missense variants from both the PBD and DBD.

Y3035S (classified with conflicting interpretations in ClinVar) was included alongside W2626C (pathogenic) to investigate the functional impact of known low-penetrance/risk allele variants using CRISPR-Select. The inclusion of additional low-penetrance/risk allele variants was limited, as only W2626C and Y3035S have been evaluated for disease penetrance or association in large clinical cohorts (24–26). Notably, while K2729N (benign) was previously reported as a risk allele in Asian populations (25), it is not considered a “risk allele” in this study owing to its high allele frequencies (BA1) among East and South Asians.

### Cells and culture conditions

Generation of the iCas9-MCF10A-*BRCA2*^+/–^ cell line used in this study has previously been described by Niu et al. (23). iCas9-MCF10A-*BRCA2*^+/–^ cells were cultured in DMEM/F-12, HEPES (Thermo Fisher Scientific, 31330038) supplemented with 5% (vol/vol) horse serum (Thermo Fisher Scientific, 26050088), 1% penicillin/streptomycin (Thermo Fisher Scientific, 15070063), 10 μg/mL insulin (Sigma-Aldrich, I1882), 20 ng/mL epidermal growth factor (PeproTech, AF-100-15), 0.5 μg/mL hydrocortisone (Sigma-Aldrich, H0888), and 100 ng/mL cholera toxin (Sigma-Aldrich, C8052). The human osteosarcoma cell line (U-2-OS), obtained from ATCC, was grown in DMEM, high glucose, GlutaMAX Supplement, pyruvate (Thermo Fisher Scientific, 31966021) supplemented with 10% (vol/vol) FBS South America, Tetracycline Free (Biowest, S181T) and 1% penicillin/streptomycin (Thermo Fisher Scientific, 15070063). Both cell lines were maintained at 37°C in a 5% CO_2_ incubator and underwent regular testing for the absence of mycoplasma contamination.

### CRISPR-Select cassette design

#### CRISPR RNA design.

CRISPR RNAs (crRNAs) were designed using the Integrated DNA Technologies (IDT) custom Alt-R CRISPR-Cas9 guide RNA design tool and further optimized to identify the best performing crRNAs. To maximize efficient incorporation of ssODNs, variants were edited with specific crRNAs based on their proximity to the Cas9 cleavage site and overall crRNA editing efficiency. A list of all the crRNAs and the respective variants edited using these crRNAs is provided in Supplemental Table 5.

#### ssODN repair templates design.

As shown in Figure 1B, for each variant, we designed 2 different ssODN repair templates containing the variant of interest (referred to as “variant ssODN”) and a synonymous internal normalization mutation (referred to as “WT′ ssODN”). To promote similar knockin efficiency, the synonymous WT′ mutation was preferentially placed at the same position or within 1–3 nucleotides from the variant of interest. For variants located in splicing motifs, the synonymous WT′ mutation was placed slightly off the variant of interest to avoid unintended splicing events. Owing to poor homology-directed repair (HDR) incorporation, for certain variants, additional silent mutations targeting the protospacer-adjacent motif or guide RNA seed region were introduced in both the variant and WT′ ssODNs to abolish guide RNA binding after successful HDR. The length of ssODN homology arms was between 41 and 45 nucleotides, and a list of all the ssODNs used in this study is provided in Supplemental Table 6.

### CRISPR-Select assay

The CRISPR-Select assay was performed as described in Niu et al. (23). Cas9 expression in iCas9-MCF10A-*BRCA2*^+/–^ cells was induced by adding 1 μg/mL doxycycline (Sigma-Aldrich, D9891) to the culture medium 24 hours before transfection of crRNA-trans-activating crRNA (tracrRNA) and ssODNs. Briefly, for a 9.6 cm^2^ well, sgRNA was prepared by mixing equimolar concentrations (75 pmol) of crRNA (10 μM, IDT) and tracrRNA (10 μM, IDT, 1072533). The sgRNA was further heated for 5 minutes at 95°C and then allowed to cool to room temperature. ssODNs were purchased from IDT as unmodified Ultramer DNA oligonucleotides (100 μM in IDTE buffer, pH 8.0). Furthermore, 10 pmol of variant and WT′ ssODNs (10 μM) together with 125 μL OptiMEM (Thermo Fisher Scientific, 31985062) were added to the sgRNA and mixed gently. Next, the sgRNA:ssODN solution was mixed with 7.5 μL Lipofectamine RNAiMAX (Thermo Fisher Scientific, 13778150) and 125 μL OptiMEM, followed by incubation at room temperature for 10 minutes. During the incubation, fresh culture medium containing 1 μg/mL doxycycline and 1 μM HDR enhancer V2 (IDT, 10007921) was added onto the cells. Upon completion of the incubation, the transfection mixture containing sgRNA:ssODN:RNAiMAX was added dropwise onto the cells and incubated for 2 days at 37°C. For other cell culture plates/dishes, the reagent volumes detailed above were adjusted according to the cell culture surface area.

On day 2, after delivery of the sgRNA:ssODN:RNAiMAX mixture, an aliquot containing >17,000 cells of the relevant cell population was isolated to estimate the variant/WT′ HDR incorporation ratio. The remainder of the cells was further split into 4 different dishes containing fresh cell culture medium with no drugs (untreated), 1 nM Talazoparib (PARPi) (MedChemExpress, HY-16106), 1 μM cisplatin (Selleck Chemicals GmbH, S1166), or 5 ng/mL MMC (Sigma-Aldrich, M5353) (Supplemental Figure 1). Cells were cultivated for an additional 10 days with frequent splitting when they became confluent. The cell culture medium (with or without the aforementioned drugs) was changed once every 3 days. During every splitting, >17,000 cells were passaged. On day 12, cells were extracted to estimate the final variant/WT′ ratio to determine the relevant pathogenicity.

#### Rationale for conducting cell survival and drug response assays.

In untreated cells, pathogenic mutations in *BRCA2* typically impair the homologous recombination (HR) repair pathway, leading to the accumulation of DNA double-strand breaks and subsequent cell death. Treatment with clinically relevant DNA-damaging agents such as PARPi, which block the repair of single-strand breaks, or agents like cisplatin and MMC, which induce DNA crosslinks, results in the formation of additional double-strand breaks. Under normal circumstances, these breaks are repaired through a BRCA2-dependent HR mechanism. However, in cells harboring pathogenic *BRCA2* mutations, the HR repair pathway is defective, thereby compromising the cell’s ability to repair the induced DNA damage. This creates a synthetic lethal effect when exposed to these agents, leading to exacerbated cell death (47). Variants exhibiting partial effects owing to their hypomorphic characteristics under untreated conditions could be investigated for their clinical relevance by utilizing these DNA-damaging agents. Although the role of drug sensitivity assays in pathogenicity estimation is still under deliberation, we decided to implement them in our variant classification. This decision was based on the findings of Boonen et al. (48) and Bouwman et al. (49), who found a higher degree of correlation between direct repeat GFP and drug sensitivity assays (PARPi and cisplatin).

### NGS library preparation and sequencing

Genomic DNA was extracted using the QIAamp DNA Mini Kit (Qiagen, 51306) as per the manufacturer’s instructions. To prepare the PCR products for amplicon NGS, 100 ng genomic DNA (corresponding to approximately 17,000 cells) was used as the template in a 2-round PCR-based approach. In the first-round PCR, the target site was amplified using target-site-specific primers containing overhangs with binding sites for the second-round barcoded primer pairs. The first-round PCR was performed in a total volume of 25 μL, containing 12.5 μL Phusion U Green Multiplex PCR Master Mix (Thermo Fisher Scientific, F564L), 0.3 μM of each primer, and template DNA. The first-round PCR was performed with an initial denaturing for 1 minute at 98°C, followed by 35 cycles of 98°C for 10 seconds, 60°C for 30 seconds (reducing the temperature by 0.1°C each cycle), 72°C for 15 seconds, and a final post-PCR extension for 5 minutes at 72°C. In the second-round PCR, 3 μL of the first-round PCR product (template) was amplified using 0.3 μM of barcoded primers and 6.25 μL Phusion U Green Multiplex PCR Master Mix. The second-round PCR was performed with an initial denaturing for 30 seconds at 98°C, followed by 8 cycles of 98°C for 10 seconds, 67°C for 30 seconds, 72°C for 15 seconds, and a final post-PCR extension for 5 minutes at 72°C. The amplicon sequencing library was prepared using the MiSeq Reagent Kit v2 (Illumina, MS-102-2002) and sequenced in a MiSeq instrument (Illumina), according to the manufacturer’s instructions. A total of at least 7,500 NGS reads per sample were used**)**. A list of all the primers used in this study is provided in Supplemental Table 7. NGS data were analyzed using the CRISPResso2 tool with minimum homology for alignment set to 80% (50).

### CRISPR-Select analysis

The counts of variant of interest (hereafter referred to as the variant), WT′, and Frameshift InDel-containing reads were determined using CRISPResso2. Among these, Frameshift InDel reads arise naturally as a result of DNA double-strand breaks induced by CRISPR-Cas9 activity.

The variant and WT′ values were utilized to assess the deleteriousness of each genetic variant under normal cell culture conditions (untreated) or in the presence of 1 nM Talazoparib (PARPi), 1 μM cisplatin, or 5 ng/mL MMC. To achieve this, as illustrated in Figure 1B and detailed in Supplemental Table 8, the variant/WT′ ratio was calculated for Day2 and Day12 (untreated and drug treated samples). These ratios were then normalized to the day 2 variant/WT′ ratio, which is set at 100%. The normalization allows the ratios for day 12 untreated and treated with PARPi, cisplatin, and MMC to reflect variant effect. Additionally, the normalized variant/WT′ ratios were averaged across biological replicates and are reported as mean ± SD in Figures 2 and 3. The normalized values for each variant were consolidated into a single plot and presented as Figure 2A (untreated), Figure 2B (PARPi), Figure 3A (cisplatin), and Figure 3B (MMC). Please refer to Supplemental Table 8 for an example of the variant/WT′ ratio analysis.

In Supplemental Figure 2, Frameshift InDel and WT′ values were used as internal controls to identify negative selection against cells with frameshift indels in the same cell culture dish, where the benign/likely benign and neutral variants were not selected against. As outlined in Supplemental Table 9, the Frameshift InDel/WT′ ratio was calculated for Day 2 and Day 12 (untreated and drug treated samples). These ratios were then normalized to the day 2 Frameshift InDel/WT′ ratio, which is set at 100%. Additionally, the normalized Frameshift InDel/WT′ ratios were averaged across biological replicates and are reported as mean ± SD in Supplemental Figure 2. This normalization enables the visualization of a strong negative selection against cells carrying frameshift indels at day 12, both untreated and treated with PARPi, cisplatin, and MMC. Please refer to Supplemental Table 9 for an example of the Frameshift InDel/WT′ ratio analysis.

### ACMG/AMP framework for classification of BRCA2-PBD variants

We used the recently established guidelines of the ClinGen *BRCA1/2* VCEP for clinical classification of the shortlisted *BRCA2*-PBD variants (Supplemental Table 3) (18). The individual ACMG/AMP clinical codes employed and their description are provided below. We initially used the original qualitative ACMG/AMP code combinations to identify the pathogenicity of VUS (19). Then, for comparison, the codes were combined following the point-based system recently proposed to simplify variant classification (51, 52).

Points for different code strengths were assigned as recommended in Tavtigian et al. (51). In particular, we used the following. For the pathogenic class, indeterminate = 0, supporting = 1, moderate = 2, strong = 4, and very strong = 8, and for the benign class: indeterminate = 0, supporting = −1, moderate = −2, strong = −4, and very strong = −8. These points were further combined to derive the final pathogenicity using the following point scale: benign, ≤−7; likely benign, −6 to −2; uncertain, −1 to 5; likely pathogenic, 6 to 9; and pathogenic, ≥10 (43, 52).

#### PS3/BS3.

The initial ACMG/AMP recommendations proposed the use of results from well-established functional assays under the strong evidence codes PS3/BS3 (19). However, the ClinGen Sequence Variant Interpretation (SVI) working group has recently recommended the use of evidence strength based on the OddsPath for each individual functional assay (please refer to Brnich et al., ref. 27, and Brnich et al., ref. 53, for more details) (27, 53). Since our assay included 9 benign/likely benign and 14 likely pathogenic/pathogenic controls with a perfect binary readout, the OddsPath for our assay was 9 for pathogenic and 0.071 for benign variants, thus allowing us to use PS3/BS3 at moderate evidence strength (27). Of note, a minimum of 19 benign and 19 pathogenic controls with a perfect binary readout (OddsPath = 19) is required to use PS3/BS3 at full strength (27). Taken together, we used the following scheme to apply ACMG/AMP codes PS3/BS3 to our VUS classification efforts: PS3-Moderate, deleterious variants in either untreated or drug sensitivity assays; PS3-Indeterminate, intermediate variants in either untreated or drug sensitivity assays, without evidence of deleteriousness; and BS3-Moderate, neutral variants in both untreated and drug sensitivity assays. PS3-Indeterminate evidence strength was used to not abate the hypomorphic variants (by conferring BS3-Moderate strength), since it would impact VUS classification under the point-based ACMG/AMP classification system.

#### PM2/BA1/BS1.

The ClinGen *BRCA1/2* VCEP recommendations were followed while assigning population frequency-based evidence strength (18). The following pathogenic and benign code strengths were applied according to gnomAD nonfounder population (non-Finnish European, African, Latino, East Asian, and South Asian) allele frequencies: BA1, filter allele frequency (FAF) >0.001; BS1, FAF >0.0001 to ≤0.001; BS1-Supporting, FAF >0.00002 to ≤0.0001; PM2-Indeterminate, minor allele frequency >0 to ≤0.00002; and PM2-Supporting, absent in controls. Though not yet recommended by the ClinGen *BRCA1/2* VCEP, we still used PM2-Indeterminate here, as evidence strength for variants with minor allele frequency >0 to ≤0.00002 was not proposed earlier.

#### PP3/BP4.

Bioinformatic predictions (54) were performed according to recommendations from the ClinGen *BRCA1/2* VCEP and SVI Splicing Subgroup (55) to identify spliceogenicity and functional consequences of all VUS included in this study, as follows: PP3, SpliceAI Δ score ≥0.2 and/or BayesDel score ≥0.30, and BP4, SpliceAI Δ score ≤0.1 and BayesDel score ≤0.18. PP3 was not applied to the D23H variant owing to the use of PVS1_Strong (RNA) code, which was assigned based on the findings from minigene splicing assay (18).

#### PS1(Splicing).

PS1(Splicing) codes were applied for the *BRCA2* exon 2 donor splice region variants (A22 = c.66A>C, A22 = c.66A>G, and A22 = c.66A>T) according to recommendations from the ClinGen *BRCA1/2* VCEP and SVI Splicing Subgroup (18, 55). PS1(Splicing) was applied at moderate strength for A22 = c.66A>C, A22 = c.66A>G, and A22 = c.66A>T based on D23Y and intervening sequence variants reported in ClinVar and LOVD (6, 56).

PS1(Splicing) was not applied to the D23H variant owing to the use of PVS1_Strong (RNA) code, which was assigned based on the findings from minigene splicing assay (18).

#### PVS1_Strong (RNA).

Based on the recommendations from ClinGen *BRCA1/2* VCEP (18), PVS1_Strong (RNA) was applied to D23H owing to the complete loss of functional transcript observed in the minigene splicing assay.

### Drug sensitivity assay

iCas9-MCF10A-*BRCA2*^+/–^ cells were seeded onto 96-well microplates (Thermo Fisher Scientific), and reverse transfection with 50 nM siRNA was performed using Lipofectamine RNAiMAX (Thermo Fisher Scientific) as per the manufacturer’s recommendation. The ON-TARGETplus Non-targeting Control Pool (UNC; Dharmacon) was used as a negative control; the oligonucleotide sequence 5′-GGAAUGUUCCCAAUAGUAG[dT][dT] (Sigma-Aldrich) was used for knockdown of BRCA2. After 24 hours, different concentrations of talazoparib, cisplatin, and MMC were added to the respective wells. At day 5, CellTiter-Glo 2.0 (Promega) was used to quantify the number of viable cells as per the manufacturer’s recommendation. Surviving fractions were calculated relative to untreated cells for each drug concentration.

### Immunoblotting and antibodies

Cells were lysed on ice in radioimmunoprecipitation assay buffer (RIPA buffer; Thermo Fisher Scientific) supplemented with 250 units of benzonase (Sigma Aldrich), 1x Halt protease and phosphatase inhibitors cocktail (Thermo Fisher Scientific), and 50 mM reducing agent (NuPAGE, Thermo Fisher Scientific). Proteins were resolved by SDS-PAGE using 4x LSB buffer (NuPAGE, Thermo Fisher Scientific) and transferred to a nitrocellulose membrane. Blocking and blotting with primary and secondary antibodies were performed in TBS-T (Thermo Fisher Scientific) supplemented with 5% skimmed milk powder (Merck Millipore). Blots were developed as per the manufacturer’s instructions using Immobilon Forte Western HRP substrate (Merck Millipore), and chemiluminescence was detected using Amersham Imager 600. Immunoblots were performed using the following antibodies: BRCA2 (catalog OP95, Merck Millipore), Vinculin (catalog V284, Merck Millipore), and anti-mouse HRP-linked (catalog 7076, Cell Signaling).

### Minigene splicing assay

WT *BRCA2* exon 2, along with flanking intronic sequences, was PCR amplified from human genomic DNA using *BRCA2*-F, 5′-GATCACCTCGAGCTCCGCCTTCAGCTCAAGACTTAAC-3′ and *BRCA2*-R 5′-GATCACCTGCAGAGCACTCCGGGGGTCCTAGAT-3′ oligonucleotides. The PCR products were treated with *XhoI* and *PstI* and cloned into the pSPL3 vector (Gibco-BRL). This pSPL3 *BRCA2*-Exon2 plasmid was used for site directed mutagenesis, to introduce *BRCA2*-Exon2-c.67G>C (D23H) mutation using BRCA2_D23H (5′-CGCTGCAACAAAGCACGTATTGACAAATTTTA-3′) and pSPL3seq_R (5′-CTACTTCTTGTGGGTTGGGGTC-3′) oligonucleotides, following a transfer-PCR approach mentioned in Erijman et al. (57). All constructs were verified by sequencing using pSPL3seq_F (5′-GAGCAGAAGACAGTGGCAATGAG-3′) oligonucleotide. Furthermore, pSPL3 (Empty), pSPL3 *BRCA2*-Exon2 (WT), and pSPL3 *BRCA2*-Exon2-c.67G>C (D23H) plasmids were transfected into U-2-OS cells using GenJet In Vitro DNA Transfection Reagent (SignaGen Laboratories, SL100489-MCF10A) as per the manufacturer’s instructions. After 2 days, cells were treated with 300 μg/mL Cycloheximide (Merck Millipore, C4859-1ML) for 4 hours to block nonsense-mediated mRNA decay. Furthermore, RNA was purified using the RNeasy Mini Kit (QIAGEN, 74104), and cDNA was synthesized using Maxima H Minus First Strand cDNA Synthesis Kit (Thermo Fisher Scientific, K1682). cDNA was amplified with Phusion U Green Multiplex PCR Master Mix (Thermo Fisher Scientific, F564L) using the primers dUSD2 (5′-TCTGAGTCACCTGGACAACC-3′) and dUSA4 (5′-ATCTCAGTGGTATTTGTGAGC-3′). PCR products were separated by electrophoresis on a 1.5% agarose gel containing ethidium bromide. Targeted NGS with pSPL3-specific primers (pSPL3-NGS-F, 5′-ACACTCTTTCCCTACACGACGCTCTTCCGATCTGAACTGCACTGTGACAAGCTG-3′ and pSPL3-NGS-R, 5′-TGACTGGAGTTCAGACGTGTGCTCTTCCGATCTCCACCTTCTGATAGGCAGCC-3′) or Sanger sequencing with dUSD2 was performed to identify the splicing patterns.

### Statistics

Assay thresholds were established using ROC graphs following guidance from Fawcett (28). The sensitivity and specificity of CRISPR-Select were estimated based on the included 9 benign/likely benign variants and 14 likely pathogenic/pathogenic variants using contingency table analysis. The 95% CIs for sensitivity and specificity were calculated using the Wilson/Brown method. Y3035S was excluded from the contingency table analysis owing to conflicting classifications in ClinVar. Data were analyzed and visualized using Microsoft Excel and GraphPad Prism 10 software.

### Study approval

As no human or animal models were used in this study, study approval was not required.

### Data availability

All supporting data from the study can be found in the Supporting Data Values file. All FASTQ files analyzed in this study have been deposited in the NCBI BioProject database under accession number PRJNA1238183.

## Author contributions

MB, CSS, and MR conceived, designed, and oversaw the study. MB and MIS carried out the experiments. MB performed the analysis. MF and CSS are the inventors of CRISPR-Select. MB and MR drafted the manuscript. BE provided clinical insights to the study and all authors contributed to critical review of the paper.

## Supplementary Material

Supplemental data

ICMJE disclosure forms

Unedited blot and gel images

Supplemental tables 1-3, 8 and 9

Supporting data values

## Version 1 04/15/2025

In-Press Preview

## Version 2 06/16/2025

Electronic publication
