# Supplementary material for: Precision screening facilitates clinical classification of BRCA2-PALB2 binding variants with benign and pathogenic functional effects
Source: J Clin Invest. 2025 Apr 15;135(12):e181879. doi: 10.1172/JCI181879 (PMC12165785; doi:10.1172/JCI181879)
Supplement: Supplemental data [file jci-135-181879-s030.pdf]

Supplemental Table 5. Sequence of crRNAs and the respective variants edited using these crRNAs

crRNA sequence	Variants edited using the specific crRNA
AAAAAATGTTGGCCTCTCTT	T10P, T10I, T10K, T10=, F11L (c.31T>C), F11V, F11C, F11L (c.33T>G), F12S
TTAAAAATTTCAAAAAATGT	E13*, E13K, E13V, E13D, I14V, I14N, I14M
AAGACACGCTGCAACAAAGC	F15L, F15C, K16R, T17I, T17=, R18H, C19R, C19Y, C19F, A22= (c.66A>C), A22= (c.66A>G), A22= (c.66A>T), D23H
TCAAACCAATTAAGACTTAT	D23G, D23V, L24V, L24F, G25*, G25R, G25E, P26S, P26R, P26L, P26=, S28G, S28N, N30D, N30H, N30S, N30K
TAGGACCAATAAGTCTTAAT	L29V, L29R, N30D, N30H, N30S, N30K, W31R, W31G, W31L, W31C, F32L (c.94T>C), F32L (c.96T>G), E33*, E33K, L35F
GCAGGTTCAGAATTATAGGG	S36Y, S36F, S37A, S37L, S37*, E38K, A39=
GGGTTTATAATCACTATAGA	H2623Y, H2623R
ATCACTATAGATGGATCATA	W2626C, I2627F
TTATTGAACTTACAGATGGG	T2722R, D2723A, D2723G
AACTCAGTATCAACAACTAC	Y3035S
CAGAAACGACAAATCCTATT	G3076V, G3076E, V3079I
AGATGGGTGGTATGCTGTTA	V2728I, K2729N
TGTTCTGAGGTGGACCTAAT	L3074=

Few variants (such as N30D, N30H, N30S and N30K) are edited using multiple crRNAs

Supplemental Table 6. List of all ssODNs used in this study

T10P

Ref: ATATCGTAGGTAAAAATGCCTATTGGATCCAAAGAGAGGCCAACATTTTTTGAAATTTTTAAGACACGCTGCAACAAAGCAGGTATTGAC
M P I G S K E R P T F F E I F K T R C N K A
MUT: ATATCGTAGGTAAAAATGCCTATTGGATCAAAAGAGAGGCCACCATTTTTTGAAATTTTTAAGACACGCTGCAACAAAGCAGGTATTGAC
M P I G S K E R P P F F E I F K T R C N K A
WT′: ATATCGTAGGTAAAAATGCCTATTGGATCAAAAGAGAGGCCAACCTTTTTTGAAATTTTTAAGACACGCTGCAACAAAGCAGGTATTGAC
M P I G S K E R P T F F E I F K T R C N K A

T10I

Ref: ATATCGTAGGTAAAAATGCCTATTGGATCCAAAGAGAGGCCAACATTTTTTGAAATTTTTAAGACACGCTGCAACAAAGCAGGTATTGAC
M P I G S K E R P T F F E I F K T R C N K A
MUT: ATATCGTAGGTAAAAATGCCTATTGGATCAAAAGAGAGGCCAATATTTTTTGAAATTTTTAAGACACGCTGCAACAAAGCAGGTATTGAC
M P I G S K E R P I F F E I F K T R C N K A
WT′: ATATCGTAGGTAAAAATGCCTATTGGATCAAAAGAGAGGCCAACCTTTTTTGAAATTTTTAAGACACGCTGCAACAAAGCAGGTATTGAC
M P I G S K E R P T F F E I F K T R C N K A

T10K

Ref: ATATCGTAGGTAAAAATGCCTATTGGATCCAAAGAGAGGCCAACATTTTTTGAAATTTTTAAGACACGCTGCAACAAAGCAGGTATTGAC
M P I G S K E R P T F F E I F K T R C N K A
MUT: ATATCGTAGGTAAAAATGCCTATTGGATCAAAAGAGAGGCCAAAATTTTTTGAAATTTTTAAGACACGCTGCAACAAAGCAGGTATTGAC
M P I G S K E R P K F F E I F K T R C N K A
WT′: ATATCGTAGGTAAAAATGCCTATTGGATCAAAAGAGAGGCCAACCTTTTTTGAAATTTTTAAGACACGCTGCAACAAAGCAGGTATTGAC
M P I G S K E R P T F F E I F K T R C N K A

T10=

```
Ref: ATATCGTAGGTAAAAATGCCTATTGGATCCAAAGAGAGGCCAACATTTTTTGAAATTTTTAAGACACGCTGCAACAAAGCAGGTATTGAC
                         M  P  I  G  S  K  E  R  P  T  F  F  E  I  F  K  T  R  C  N  K  A
MUT: ATATCGTAGGTAAAAATGCCTATTGGATCAAAAGAGAGGCCAACGTTTTTTGAAATTTTTAAGACACGCTGCAACAAAGCAGGTATTGAC
                         M  P  I  G  S  K  E  R  P  T  F  F  E  I  F  K  T  R  C  N  K  A
WT': ATATCGTAGGTAAAAATGCCTATTGGATCAAAAGAGAGGCCAACCTTTTTTGAAATTTTTAAGACACGCTGCAACAAAGCAGGTATTGAC
                         M  P  I  G  S  K  E  R  P  T  F  F  E  I  F  K  T  R  C  N  K  A
```

F11L (c.31T>C)

```
Ref: ATCGTAGGTAAAAATGCCTATTGGATCCAAAGAGAGGCCAACATTTTTTGAAATTTTTAAGACACGCTGCAACAAAGCAGGTATTGACAA
                       M  P  I  G  S  K  E  R  P  T  F  F  E  I  F  K  T  R  C  N  K  A
MUT: ATCGTAGGTAAAAATGCCTATTGGATCAAAAGAGAGGCCAACACTTTTTGAAATTTTTAAGACACGCTGCAACAAAGCAGGTATTGACAA
                       M  P  I  G  S  K  E  R  P  T  L  F  E  I  F  K  T  R  C  N  K  A
WT': ATCGTAGGTAAAAATGCCTATTGGATCAAAAGAGAGGCCAACATTCTTTGAAATTTTTAAGACACGCTGCAACAAAGCAGGTATTGACAA
                       M  P  I  G  S  K  E  R  P  T  F  F  E  I  F  K  T  R  C  N  K  A
```

F11V

```
Ref: ATCGTAGGTAAAAATGCCTATTGGATCCAAAGAGAGGCCAACATTTTTTGAAATTTTTAAGACACGCTGCAACAAAGCAGGTATTGACAA
                       M  P  I  G  S  K  E  R  P  T  F  F  E  I  F  K  T  R  C  N  K  A
MUT: ATCGTAGGTAAAAATGCCTATTGGATCAAAAGAGAGGCCAACAGTTTTTGAAATTTTTAAGACACGCTGCAACAAAGCAGGTATTGACAA
                       M  P  I  G  S  K  E  R  P  T  V  F  E  I  F  K  T  R  C  N  K  A
WT': ATCGTAGGTAAAAATGCCTATTGGATCAAAAGAGAGGCCAACATTCTTTGAAATTTTTAAGACACGCTGCAACAAAGCAGGTATTGACAA
                       M  P  I  G  S  K  E  R  P  T  F  F  E  I  F  K  T  R  C  N  K  A
```

F11C

```
Ref: ATCGTAGGTAAAAATGCCTATTGGATCCAAAGAGAGGCCAACATTTTTTGAAATTTTTAAGACACGCTGCAACAAAGCAGGTATTGACAA
                       M  P  I  G  S  K  E  R  P  T  F  F  E  I  F  K  T  R  C  N  K  A
MUT: ATCGTAGGTAAAAATGCCTATTGGATCAAAAGAGAGGCCAACATGTTTTGAAATTTTTAAGACACGCTGCAACAAAGCAGGTATTGACAA
                       M  P  I  G  S  K  E  R  P  T  C  F  E  I  F  K  T  R  C  N  K  A
WT': ATCGTAGGTAAAAATGCCTATTGGATCAAAAGAGAGGCCAACATTCTTTGAAATTTTTAAGACACGCTGCAACAAAGCAGGTATTGACAA
                       M  P  I  G  S  K  E  R  P  T  F  F  E  I  F  K  T  R  C  N  K  A
```

F11L (c.33T>G)

```
Ref: ATCGTAGGTAAAAATGCCTATTGGATCCAAAGAGAGGCCAACATTTTTTGAAATTTTTAAGACACGCTGCAACAAAGCAGGTATTGACAA
                       M  P  I  G  S  K  E  R  P  T  F  F  E  I  F  K  T  R  C  N  K  A
MUT: ATCGTAGGTAAAAATGCCTATTGGATCAAAAGAGAGGCCAACATTGTTTGAAATTTTTAAGACACGCTGCAACAAAGCAGGTATTGACAA
                       M  P  I  G  S  K  E  R  P  T  L  F  E  I  F  K  T  R  C  N  K  A
WT': ATCGTAGGTAAAAATGCCTATTGGATCAAAAGAGAGGCCAACATTCTTTGAAATTTTTAAGACACGCTGCAACAAAGCAGGTATTGACAA
                       M  P  I  G  S  K  E  R  P  T  F  F  E  I  F  K  T  R  C  N  K  A
```

F12S

```
Ref: TAGGTAAAAATGCCTATTGGATCCAAAGAGAGGCCAACATTTTTTGAAATTTTTAAGACACGCTGCAACAAAGCAGGTATTGACAAATTT
                  M  P  I  G  S  K  E  R  P  T  F  F  E  I  F  K  T  R  C  N  K  A
MUT: TAGGTAAAAATGCCTATTGGATCAAAAGAGAGGCCAACATTTTCTGAAATTTTTAAGACACGCTGCAACAAAGCAGGTATTGACAAATTT
                  M  P  I  G  S  K  E  R  P  T  F  S  E  I  F  K  T  R  C  N  K  A
WT': TAGGTAAAAATGCCTATTGGATCAAAAGAGAGGCCAACATTTTTCGAAATTTTTAAGACACGCTGCAACAAAGCAGGTATTGACAAATTT
                  M  P  I  G  S  K  E  R  P  T  F  F  E  I  F  K  T  R  C  N  K  A
```

E13*

```
Ref: TAGGTAAAAATGCCTATTGGATCCAAAGAGAGGCCAACATTTTTTGAAATTTTTAAGACACGCTGCAACAAAGCAGGTATTGACAAATTT
                 M  P  I  G  S  K  E  R  P  T  F  F  E  I  F  K  T  R  C  N  K  A
MUT: TAGGTAAAAATGCCTATTGGATCCAAAGAGAGGCCAACATTCTTCTAAATTTTTAAGACACGCTGCAACAAAGCAGGTATTGACAAATTT
                 M  P  I  G  S  K  E  R  P  T  F  F  -  I  F  K  T  R  C  N  K  A
WT': TAGGTAAAAATGCCTATTGGATCCAAAGAGAGGCCAACATTCTTCGAGATTTTTAAGACACGCTGCAACAAAGCAGGTATTGACAAATTT
                 M  P  I  G  S  K  E  R  P  T  F  F  E  I  F  K  T  R  C  N  K  A
```

E13K

```
Ref: TAGGTAAAAATGCCTATTGGATCCAAAGAGAGGCCAACATTTTTTGAAATTTTTAAGACACGCTGCAACAAAGCAGGTATTGACAAATTT
                 M  P  I  G  S  K  E  R  P  T  F  F  E  I  F  K  T  R  C  N  K  A
MUT: TAGGTAAAAATGCCTATTGGATCCAAAGAGAGGCCAACATTCTTCAAAATTTTTAAGACACGCTGCAACAAAGCAGGTATTGACAAATTT
                 M  P  I  G  S  K  E  R  P  T  F  F  K  I  F  K  T  R  C  N  K  A
WT': TAGGTAAAAATGCCTATTGGATCCAAAGAGAGGCCAACATTCTTCGAGATTTTTAAGACACGCTGCAACAAAGCAGGTATTGACAAATTT
                 M  P  I  G  S  K  E  R  P  T  F  F  E  I  F  K  T  R  C  N  K  A
```

E13V

```
Ref: TAGGTAAAAATGCCTATTGGATCCAAAGAGAGGCCAACATTTTTTGAAATTTTTAAGACACGCTGCAACAAAGCAGGTATTGACAAATTT
                 M  P  I  G  S  K  E  R  P  T  F  F  E  I  F  K  T  R  C  N  K  A
MUT: TAGGTAAAAATGCCTATTGGATCCAAAGAGAGGCCAACATTCTTCGTTATTTTTAAGACACGCTGCAACAAAGCAGGTATTGACAAATTT
                 M  P  I  G  S  K  E  R  P  T  F  F  V  I  F  K  T  R  C  N  K  A
WT': TAGGTAAAAATGCCTATTGGATCCAAAGAGAGGCCAACATTCTTCGAGATTTTTAAGACACGCTGCAACAAAGCAGGTATTGACAAATTT
                 M  P  I  G  S  K  E  R  P  T  F  F  E  I  F  K  T  R  C  N  K  A
```

E13D

```
Ref: TAGGTAAAAATGCCTATTGGATCCAAAGAGAGGCCAACATTTTTTGAAATTTTTAAGACACGCTGCAACAAAGCAGGTATTGACAAATTT
                 M  P  I  G  S  K  E  R  P  T  F  F  E  I  F  K  T  R  C  N  K  A
MUT: TAGGTAAAAATGCCTATTGGATCCAAAGAGAGGCCAACATTCTTCGACATTTTTAAGACACGCTGCAACAAAGCAGGTATTGACAAATTT
                 M  P  I  G  S  K  E  R  P  T  F  F  D  I  F  K  T  R  C  N  K  A
WT': TAGGTAAAAATGCCTATTGGATCCAAAGAGAGGCCAACATTCTTCGAGATTTTTAAGACACGCTGCAACAAAGCAGGTATTGACAAATTT
                 M  P  I  G  S  K  E  R  P  T  F  F  E  I  F  K  T  R  C  N  K  A
```

I14V

```
Ref: TAAAAATGCCTATTGGATCCAAAGAGAGGCCAACATTTTTTGAAATTTTTAAGACACGCTGCAACAAAGCAGGTATTGACAAATTTTATAT
         M  P  I  G  S  K  E  R  P  T  F  F  E  I  F  K  T  R  C  N  K  A
MUT: TAAAAATGCCTATTGGATCCAAAGAGAGGCCAACATTTTTTGAAGTTTTTAAGACACGCTGCAACAAAGCAGGTATTGACAAATTTTATAT
         M  P  I  G  S  K  E  R  P  T  F  F  E  V  F  K  T  R  C  N  K  A
WT': TAAAAATGCCTATTGGATCCAAAGAGAGGCCAACATTTTTTGAAATCTTTAAGACACGCTGCAACAAAGCAGGTATTGACAAATTTTATAT
         M  P  I  G  S  K  E  R  P  T  F  F  E  I  F  K  T  R  C  N  K  A
```

I14N

```
Ref: TAAAAATGCCTATTGGATCCAAAGAGAGGCCAACATTTTTTGAAATTTTTAAGACACGCTGCAACAAAGCAGGTATTGACAAATTTTATAT
         M  P  I  G  S  K  E  R  P  T  F  F  E  I  F  K  T  R  C  N  K  A
MUT: TAAAAATGCCTATTGGATCCAAAGAGAGGCCAACATTTTTTGAAAATTTTAAGACACGCTGCAACAAAGCAGGTATTGACAAATTTTATAT
         M  P  I  G  S  K  E  R  P  T  F  F  E  N  F  K  T  R  C  N  K  A
WT': TAAAAATGCCTATTGGATCCAAAGAGAGGCCAACATTTTTTGAAATCTTTAAGACACGCTGCAACAAAGCAGGTATTGACAAATTTTATAT
         M  P  I  G  S  K  E  R  P  T  F  F  E  I  F  K  T  R  C  N  K  A
```

I14M

Ref: TAAAAATGCCTATTGGATCCAAAGAGAGG**CCA**ACATTTTTTGAAATTTTTAAGACACGCTGCAACAAAGCAGGTATTGACAAATTTTATAT
M P I G S K E R P T F F E I F K T R C N K A
MUT: TAAAAATGCCTATTGGATCCAAAGAGAGG**CCA**ACATTTTTTGAAATGTTTAAGACACGCTGCAACAAAGCAGGTATTGACAAATTTTATAT
M P I G S K E R P T F F E M F K T R C N K A
WT′: TAAAAATGCCTATTGGATCCAAAGAGAGG**CCA**ACATTTTTTGAAATCTTTAAGACACGCTGCAACAAAGCAGGTATTGACAAATTTTATAT
M P I G S K E R P T F F E I F K T R C N K A

F15L

Ref: AAATGCCTATTGGATCCAAAGAGAGGCCAACATTTTTTGAAATTTTTAAGACACGCTGCAACAAAGC**AGG**TATTGACAAATTTTATATAA
M P I G S K E R P T F F E I F K T R C N K A
MUT: AAATGCCTATTGGATCCAAAGAGAGGCCAACATTTTTTGAAATTCTTAAGACACGATGCAATAAAGC**AGG**TATTGACAAATTTTATATAA
M P I G S K E R P T F F E I L K T R C N K A
WT′: AAATGCCTATTGGATCCAAAGAGAGGCCAACATTTTTTGAAATTTTCAAGACACGATGCAATAAAGC**AGG**TATTGACAAATTTTATATAA
M P I G S K E R P T F F E I F K T R C N K A

F15C

Ref: AAATGCCTATTGGATCCAAAGAGAGGCCAACATTTTTTGAAATTTTTAAGACACGCTGCAACAAAGC**AGG**TATTGACAAATTTTATATAA
M P I G S K E R P T F F E I F K T R C N K A
MUT: AAATGCCTATTGGATCCAAAGAGAGGCCAACATTTTTTGAAATTTGTAAGACACGATGCAATAAAGC**AGG**TATTGACAAATTTTATATAA
M P I G S K E R P T F F E I C K T R C N K A
WT′: AAATGCCTATTGGATCCAAAGAGAGGCCAACATTTTTTGAAATTTTCAAGACACGATGCAATAAAGC**AGG**TATTGACAAATTTTATATAA
M P I G S K E R P T F F E I F K T R C N K A

K16R

Ref: TGCCTATTGGATCCAAAGAGAGGCCAACATTTTTTGAAATTTTTAAGACACGCTGCAACAAAGC**AGG**TATTGACAAATTTTATATAACTT
P I G S K E R P T F F E I F K T R C N K A
MUT: TGCCTATTGGATCCAAAGAGAGGCCAACATTTTTTGAAATTTTTAGGACACGATGCAATAAAGC**AGG**TATTGACAAATTTTATATAACTT
P I G S K E R P T F F E I F R T R C N K A
WT′: TGCCTATTGGATCCAAAGAGAGGCCAACATTTTTTGAAATTTTTAAAACACGATGCAATAAAGC**AGG**TATTGACAAATTTTATATAACTT
P I G S K E R P T F F E I F K T R C N K A

T17I

Ref: TATTGGATCCAAAGAGAGGCCAACATTTTTTGAAATTTTTAAGACACGCTGCAACAAAGC**AGG**TATTGACAAATTTTATATAACTTTATA
I G S K E R P T F F E I F K T R C N K A
MUT: TATTGGATCCAAAGAGAGGCCAACATTTTTTGAAATTTTTAAGATACGATGCAATAAAGC**AGG**TATTGACAAATTTTATATAACTTTATA
I G S K E R P T F F E I F K I R C N K A
WT′: TATTGGATCCAAAGAGAGGCCAACATTTTTTGAAATTTTTAAGACCCGATGCAATAAAGC**AGG**TATTGACAAATTTTATATAACTTTATA
I G S K E R P T F F E I F K T R C N K A

T17=

Ref: TATTGGATCCAAAGAGAGGCCAACATTTTTTGAAATTTTTAAGACACGCTGCAACAAAGC**AGG**TATTGACAAATTTTATATAACTTTATA
I G S K E R P T F F E I F K T R C N K A
MUT: TATTGGATCCAAAGAGAGGCCAACATTTTTTGAAATTTTTAAGACGCGATGCAATAAAGC**AGG**TATTGACAAATTTTATATAACTTTATA
I G S K E R P T F F E I F K T R C N K A
WT′: TATTGGATCCAAAGAGAGGCCAACATTTTTTGAAATTTTTAAGACCCGATGCAATAAAGC**AGG**TATTGACAAATTTTATATAACTTTATA
I G S K E R P T F F E I F K T R C N K A

R18H

```
Ref: TTGGATCCAAAGAGAGGCCAACATTTTTTGAAATTTTTAAGACACGCTGCAACAAAGCAGGTATTGACAAATTTTATATAACTTTATAAA
         G   S   K   E   R   P   T   F   F   E   I   F   K   T   R   C   N   K   A
MUT: TTGGATCCAAAGAGAGGCCAACATTTTTTGAAATTTTTAAGACACACTGCAATAAAGCAGGTATTGACAAATTTTATATAACTTTATAAA
         G   S   K   E   R   P   T   F   F   E   I   F   K   T   H   C   N   K   A
WT': TTGGATCCAAAGAGAGGCCAACATTTTTTGAAATTTTTAAGACACGATGCAATAAAGCAGGTATTGACAAATTTTATATAACTTTATAAA
         G   S   K   E   R   P   T   F   F   E   I   F   K   T   R   C   N   K   A
```

C19R

```
Ref: GATCCAAAGAGAGGCCAACATTTTTTGAAATTTTTAAGACACGCTGCAACAAAGCAGGTATTGACAAATTTTATATAACTTTATAAATTA
         S   K   E   R   P   T   F   F   E   I   F   K   T   R   C   N   K   A
MUT: GATCCAAAGAGAGGCCAACATTTTTTGAAATTTTTAAGACACGACGCAATAAAGCAGGTATTGACAAATTTTATATAACTTTATAAATTA
         S   K   E   R   P   T   F   F   E   I   F   K   T   R   R   N   K   A
WT': GATCCAAAGAGAGGCCAACATTTTTTGAAATTTTTAAGACACGATGTAATAAAGCAGGTATTGACAAATTTTATATAACTTTATAAATTA
         S   K   E   R   P   T   F   F   E   I   F   K   T   R   C   N   K   A
```

C19Y

```
Ref: GATCCAAAGAGAGGCCAACATTTTTTGAAATTTTTAAGACACGCTGCAACAAAGCAGGTATTGACAAATTTTATATAACTTTATAAATTA
         S   K   E   R   P   T   F   F   E   I   F   K   T   R   C   N   K   A
MUT: GATCCAAAGAGAGGCCAACATTTTTTGAAATTTTTAAGACACGATACAATAAAGCAGGTATTGACAAATTTTATATAACTTTATAAATTA
         S   K   E   R   P   T   F   F   E   I   F   K   T   R   Y   N   K   A
WT': GATCCAAAGAGAGGCCAACATTTTTTGAAATTTTTAAGACACGATGTAATAAAGCAGGTATTGACAAATTTTATATAACTTTATAAATTA
         S   K   E   R   P   T   F   F   E   I   F   K   T   R   C   N   K   A
```

C19F

```
Ref: GATCCAAAGAGAGGCCAACATTTTTTGAAATTTTTAAGACACGCTGCAACAAAGCAGGTATTGACAAATTTTATATAACTTTATAAATTA
         S   K   E   R   P   T   F   F   E   I   F   K   T   R   C   N   K   A
MUT: GATCCAAAGAGAGGCCAACATTTTTTGAAATTTTTAAGACACGATTCAATAAAGCAGGTATTGACAAATTTTATATAACTTTATAAATTA
         S   K   E   R   P   T   F   F   E   I   F   K   T   R   F   N   K   A
WT': GATCCAAAGAGAGGCCAACATTTTTTGAAATTTTTAAGACACGATGTAATAAAGCAGGTATTGACAAATTTTATATAACTTTATAAATTA
         S   K   E   R   P   T   F   F   E   I   F   K   T   R   C   N   K   A
```

A22= (c.66A>C)

```
Ref: AGAGGCCAACATTTTTTGAAATTTTTAAGACACGCTGCAACAAAGCAGGTATTGACAAATTTTATATAACTTTATAAATTACACCGAGAA
         R   P   T   F   F   E   I   F   K   T   R   C   N   K   A
MUT: AGAGGCCAACATTTTTTGAAATTTTTAAGACACGCTGCAATAAAGCCGGTATTGACAAATTTTATATAACTTTATAAATTACACCGAGAA
         R   P   T   F   F   E   I   F   K   T   R   C   N   K   A
WT': AGAGGCCAACATTTTTTGAAATTTTTAAGACACGCTGCAACAAGGCAGGTATTGACAAATTTTATATAACTTTATAAATTACACCGAGAA
         R   P   T   F   F   E   I   F   K   T   R   C   N   K   A
```

A22= (c.66A>G)

```
Ref: AGAGGCCAACATTTTTTGAAATTTTTAAGACACGCTGCAACAAAGCAGGTATTGACAAATTTTATATAACTTTATAAATTACACCGAGAA
         R   P   T   F   F   E   I   F   K   T   R   C   N   K   A
MUT: AGAGGCCAACATTTTTTGAAATTTTTAAGACACGCTGCAATAAAGCGGGTATTGACAAATTTTATATAACTTTATAAATTACACCGAGAA
         R   P   T   F   F   E   I   F   K   T   R   C   N   K   A
WT': AGAGGCCAACATTTTTTGAAATTTTTAAGACACGCTGTAACAAAGCAGGTATTGACAAATTTTATATAACTTTATAAATTACACCGAGAA
         R   P   T   F   F   E   I   F   K   T   R   C   N   K   A
```

A22= (c.66A>T)

```
Ref: AGAGGCCAACATTTTTTGAAATTTTTAAGACACGCTGCAACAAAGCAGGTATTGACAAATTTTATATAACTTTATAAATTACACCGAGAA
        R  P  T  F  F  E  I  F  K  T  R  C  N  K  A
MUT: AGAGGCCAACATTTTTTGAAATTTTTAAGACACGCTGCAATAAAGCTGGTATTGACAAATTTTATATAACTTTATAAATTACACCGAGAA
        R  P  T  F  F  E  I  F  K  T  R  C  N  K  A
WT': AGAGGCCAACATTTTTTGAAATTTTTAAGACACGCTGCAACAAGGCAGGTATTGACAAATTTTATATAACTTTATAAATTACACCGAGAA
        R  P  T  F  F  E  I  F  K  T  R  C  N  K  A
```

D23H – ssODN pair #1

```
Ref: AGAGGCCAACATTTTTTGAAATTTTTAAGACACGCTGCAACAAAGCAGGTATTGACAAATTTTATATAACTTTATAAATTACACCGAGAA
        R  P  T  F  F  E  I  F  K  T  R  C  N  K  A
MUT: AGAGGCCAACATTTTTTGAAATTTTTAAGACACGATGCAATAAAGCACGTATTGACAAATTTTATATAACTTTATAAATTACACCGAGAA
        R  P  T  F  F  E  I  F  K  T  R  C  N  K  A
WT': AGAGGCCAACATTTTTTGAAATTTTTAAGACACGATGCAATAAAGCAGGTATTGACAAATTTTATATAACTTTATAAATTACACCGAGAA
        R  P  T  F  F  E  I  F  K  T  R  C  N  K  A
```

D23H – ssODN pair #2

```
Ref: AGAGGCCAACATTTTTTGAAATTTTTAAGACACGCTGCAACAAAGCAGGTATTGACAAATTTTATATAACTTTATAAATTACACCGAGAA
        R  P  T  F  F  E  I  F  K  T  R  C  N  K  A
MUT: AGAGGCCAACATTTTTTGAAATTTTTAAGACACGCTGCAACAAAGCACGTATTGACAAATTTTATATAACTTTATAAATTACACCGAGAA
        R  P  T  F  F  E  I  F  K  T  R  C  N  K  A
WT': AGAGGCCAACATTTTTTGAAATTTTTAAGACACGCTGCAACAAGGCAGGTATTGACAAATTTTATATAACTTTATAAATTACACCGAGAA
        R  P  T  F  F  E  I  F  K  T  R  C  N  K  A
```

D23G

```
Ref: TCACTGGTTAAAACTAAGGTGGGATTTTTTTTTTAAATAGATTTAGGACCAATAAGTCTTAATTGGTTTGAAGAACTTTCT
                                             L  G  P  I  S  L  N  W  F  E  E  L  S
MUT: TCACTGGTTAAAACTAAGGTGGGATTTTTTTTTTAAATAGGTTTAGGACCAATAAGTCTTAATTGGTTTGAAGAACTTTCT
                                             L  G  P  I  S  L  N  W  F  E  E  L  S
WT': TCACTGGTTAAAACTAAGGTGGGATTTTTTTTTTAAATAGATTTAGGGCCAATAAGTCTTAATTGGTTTGAAGAACTTTCT
                                             L  G  P  I  S  L  N  W  F  E  E  L  S
```

D23V

```
Ref: TCACTGGTTAAAACTAAGGTGGGATTTTTTTTTTAAATAGATTTAGGACCAATAAGTCTTAATTGGTTTGAAGAACTTTCT
                                             L  G  P  I  S  L  N  W  F  E  E  L  S
MUT: TCACTGGTTAAAACTAAGGTGGGATTTTTTTTTTAAATAGTTTTAGGACCAATAAGTCTTAATTGGTTTGAAGAACTTTCT
                                             L  G  P  I  S  L  N  W  F  E  E  L  S
WT': TCACTGGTTAAAACTAAGGTGGGATTTTTTTTTTAAATAGATTTAGGGCCAATAAGTCTTAATTGGTTTGAAGAACTTTCT
                                             L  G  P  I  S  L  N  W  F  E  E  L  S
```

L24V

```
Ref: ACTGGTTAAAACTAAGGTGGGATTTTTTTTTTAAATAGATTTAGGACCAATAAGTCTTAATTGGTTTGAAGAACTTTCTTC
                                           L  G  P  I  S  L  N  W  F  E  E  L  S
MUT: ACTGGTTAAAACTAAGGTGGGATTTTTTTTTTAAATAGATGTAGGACCAATAAGTCTTAATTGGTTTGAAGAACTTTCTTC
                                           V  G  P  I  S  L  N  W  F  E  E  L  S
WT': ACTGGTTAAAACTAAGGTGGGATTTTTTTTTTAAATAGATTTGGGACCAATAAGTCTTAATTGGTTTGAAGAACTTTCTTC
                                           L  G  P  I  S  L  N  W  F  E  E  L  S
```

L24F

```
Ref: ACTGGTTAAAACTAAGGTGGGATTTTTTTTTTAAATAGATTTAGGACCAATAAGTCTTAATTGGTTTGAAGAACTTTCTTC
                                             L  G  P  I  S  L  N  W  F  E  E  L  S
MUT: ACTGGTTAAAACTAAGGTGGGATTTTTTTTTTAAATAGATTTTGGACCAATAAGTCTTAATTGGTTTGAAGAACTTTCTTC
                                             F  G  P  I  S  L  N  W  F  E  E  L  S
WT': ACTGGTTAAAACTAAGGTGGGATTTTTTTTTTAAATAGATTTGGGACCAATAAGTCTTAATTGGTTTGAAGAACTTTCTTC
                                             L  G  P  I  S  L  N  W  F  E  E  L  S
```

G25*

```
Ref: GGTTAAAACTAAGGTGGGATTTTTTTTTTAAATAGATTTAGGACCAATAAGTCTTAATTGGTTTGAAGAACTTTCTTCAGA
                                          L  G  P  I  S  L  N  W  F  E  E  L  S  S
MUT: GGTTAAAACTAAGGTGGGATTTTTTTTTTAAATAGATTTATGACCAATTAGTCTTAATTGGTTTGAAGAACTTTCTTCAGA
                                          L  -  P  I  S  L  N  W  F  E  E  L  S  S
WT': GGTTAAAACTAAGGTGGGATTTTTTTTTTAAATAGATTTAGGTCCAATTAGTCTTAATTGGTTTGAAGAACTTTCTTCAGA
                                          L  G  P  I  S  L  N  W  F  E  E  L  S  S
```

G25R

```
Ref: GGTTAAAACTAAGGTGGGATTTTTTTTTTAAATAGATTTAGGACCAATAAGTCTTAATTGGTTTGAAGAACTTTCTTCAGA
                                          L  G  P  I  S  L  N  W  F  E  E  L  S  S
MUT: GGTTAAAACTAAGGTGGGATTTTTTTTTTAAATAGATTTAAGACCAATTAGTCTTAATTGGTTTGAAGAACTTTCTTCAGA
                                          L  R  P  I  S  L  N  W  F  E  E  L  S  S
WT': GGTTAAAACTAAGGTGGGATTTTTTTTTTAAATAGATTTAGGTCCAATTAGTCTTAATTGGTTTGAAGAACTTTCTTCAGA
                                          L  G  P  I  S  L  N  W  F  E  E  L  S  S
```

G25E

```
Ref: GGTTAAAACTAAGGTGGGATTTTTTTTTTAAATAGATTTAGGACCAATAAGTCTTAATTGGTTTGAAGAACTTTCTTCAGA
                                          L  G  P  I  S  L  N  W  F  E  E  L  S  S
MUT: GGTTAAAACTAAGGTGGGATTTTTTTTTTAAATAGATTTAGAACCAATTAGTCTTAATTGGTTTGAAGAACTTTCTTCAGA
                                          L  E  P  I  S  L  N  W  F  E  E  L  S  S
WT': GGTTAAAACTAAGGTGGGATTTTTTTTTTAAATAGATTTAGGTCCAATTAGTCTTAATTGGTTTGAAGAACTTTCTTCAGA
                                          L  G  P  I  S  L  N  W  F  E  E  L  S  S
```

P26S

```
Ref: AAAACTAAGGTGGGATTTTTTTTTTAAATAGATTTAGGACCAATAAGTCTTAATTGGTTTGAAGAACTTTCTTCAGAAGCT
                                      L  G  P  I  S  L  N  W  F  E  E  L  S  S  E  A
MUT: AAAACTAAGGTGGGATTTTTTTTTTAAATAGATTTAGGATCAATTAGTCTGAATTGGTTTGAAGAACTTTCTTCAGAAGCT
                                      L  G  S  I  S  L  N  W  F  E  E  L  S  S  E  A
WT': AAAACTAAGGTGGGATTTTTTTTTTAAATAGATTTAGGACCTATTAGTCTGAATTGGTTTGAAGAACTTTCTTCAGAAGCT
                                      L  G  P  I  S  L  N  W  F  E  E  L  S  S  E  A
```

P26R

```
Ref: AAAACTAAGGTGGGATTTTTTTTTTAAATAGATTTAGGACCAATAAGTCTTAATTGGTTTGAAGAACTTTCTTCAGAAGCT
                                      L  G  P  I  S  L  N  W  F  E  E  L  S  S  E  A
MUT: AAAACTAAGGTGGGATTTTTTTTTTAAATAGATTTAGGACGAATTAGTCTGAATTGGTTTGAAGAACTTTCTTCAGAAGCT
                                      L  G  R  I  S  L  N  W  F  E  E  L  S  S  E  A
WT': AAAACTAAGGTGGGATTTTTTTTTTAAATAGATTTAGGACCTATTAGTCTGAATTGGTTTGAAGAACTTTCTTCAGAAGCT
                                      L  G  P  I  S  L  N  W  F  E  E  L  S  S  E  A
```

P26L

```
Ref: AAAACTAAGGTGGGATTTTTTTTTTAAATAGATTTAGGACCAATAAGTCTTAATTGGTTTGAAGAACTTTCTTCAGAAGCT
```

L G P I S L N W F E E L S S E A

MUT: AAAACTAAGGTGGGATTTTTTTTTTAAATAGATTTAGGACTAATTAGTCTGAATTGGTTTGAAGAACTTTCTTCAGAAGCT

L G L I S L N W F E E L S S E A

WT′: AAAACTAAGGTGGGATTTTTTTTTTAAATAGATTTAGGACCTATTAGTCTGAATTGGTTTGAAGAACTTTCTTCAGAAGCT

L G P I S L N W F E E L S S E A

P26=

Ref: AAAACTAAGGTGGGATTTTTTTTTTAAATAGATTTAGGACCAATAAGTCTTAATTGGTTTGAAGAACTTTCTTCAGAAGCT

L G P I S L N W F E E L S S E A

MUT: AAAACTAAGGTGGGATTTTTTTTTTAAATAGATTTAGGACCGATTAGTCTGAATTGGTTTGAAGAACTTTCTTCAGAAGCT

L G P I S L N W F E E L S S E A

WT′: AAAACTAAGGTGGGATTTTTTTTTTAAATAGATTTAGGACCTATTAGTCTGAATTGGTTTGAAGAACTTTCTTCAGAAGCT

L G P I S L N W F E E L S S E A

S28G – ssODN pair #1

Ref: TAAGGTGGGATTTTTTTTTTAAATAGATTTAGGACCAATAAGTCTTAATTGGTTTGAAGAACTTTCTTCAGAAGCTCCACC

L G P I S L N W F E E L S S E A P

MUT: TAAGGTGGGATTTTTTTTTTAAATAGATTTAGGACCAATTGGTCTGAATTGGTTTGAAGAACTTTCTTCAGAAGCTCCACC

L G P I G L N W F E E L S S E A P

WT′: TAAGGTGGGATTTTTTTTTTAAATAGATTTAGGACCAATTAGCCTGAATTGGTTTGAAGAACTTTCTTCAGAAGCTCCACC

L G P I S L N W F E E L S S E A P

S28G – ssODN pair #2

Ref: TAAGGTGGGATTTTTTTTTTAAATAGATTTAGGACCAATAAGTCTTAATTGGTTTGAAGAACTTTCTTCAGAAGCTCCACC

L G P I S L N W F E E L S S E A P

MUT: TAAGGTGGGATTTTTTTTTTAAATAGATTTAGGACCAATAGGTCTTAATTGGTTTGAAGAACTTTCTTCAGAAGCTCCACC

L G P I G L N W F E E L S S E A P

WT′: TAAGGTGGGATTTTTTTTTTAAATAGATTTAGGACCAATAAGCCTTAATTGGTTTGAAGAACTTTCTTCAGAAGCTCCACC

L G P I S L N W F E E L S S E A P

S28N

Ref: TAAGGTGGGATTTTTTTTTTAAATAGATTTAGGACCAATAAGTCTTAATTGGTTTGAAGAACTTTCTTCAGAAGCTCCACC

L G P I S L N W F E E L S S E A P

MUT: TAAGGTGGGATTTTTTTTTTAAATAGATTTAGGACCAATTAATCTGAATTGGTTTGAAGAACTTTCTTCAGAAGCTCCACC

L G P I N L N W F E E L S S E A P

WT′: TAAGGTGGGATTTTTTTTTTAAATAGATTTAGGACCAATTAGCCTGAATTGGTTTGAAGAACTTTCTTCAGAAGCTCCACC

L G P I S L N W F E E L S S E A P

L29V

Ref: GGTGGGATTTTTTTTTTAAATAGATTTAGGACCAATAAGTCTTAATTGGTTTGAAGAACTTTCTTCAGAAGCTCCACCCTA

L G P I S L N W F E E L S S E A P P

MUT: GGTGGGATTTTTTTTTTAAATAGATTTAGGACCAATAAGCGTTAATTGGTTTGAAGAACTTTCTTCAGAAGCTCCACCCTA

L G P I S V N W F E E L S S E A P P

WT′: GGTGGGATTTTTTTTTTAAATAGATTTAGGACCAATAAGCCTGAATTGGTTTGAAGAACTTTCTTCAGAAGCTCCACCCTA

L G P I S L N W F E E L S S E A P P

L29R

Ref: GGTGGGATTTTTTTTTTAAATAGATTTAGGACCAATAAGTCTTAATTGGTTTGAAGAACTTTCTTCAGAAGCTCCACCCTA

L G P I S L N W F E E L S S E A P P

MUT: GGTGGGATTTTTTTTTTAAATAGATTTAGGACCAATAAGCCGTAATTGGTTTGAAGAACTTTCTTCAGAAGCTCCACCCTA

L G P I S R N W F E E L S S E A P P

```
WT': GGTGGGATTTTTTTTTTAAATAGATTTAGGACCAATAAGCCTGAATTGGTTTGAAGAACTTTCTTCAGAAGCTCCACCCTA
                                          L  G  P  I  S  L  N  W  F  E  E  L  S  S  E  A  P  P
```

N30D – ssODN pair #1

```
Ref: GGATTTTTTTTTTAAATAGATTTAGGACCAATAAGTCTTAATTGGTTTGAAGAACTTTCTTCAGAAGCTCCACCCTATAAT
                                      L  G  P  I  S  L  N  W  F  E  E  L  S  S  E  A  P  P  Y  N
MUT: GGATTTTTTTTTTAAATAGATTTAGGACCAATAAGCCTTGATTGGTTTGAAGAACTTTCTTCAGAAGCTCCACCCTATAAT
                                      L  G  P  I  S  L  D  W  F  E  E  L  S  S  E  A  P  P  Y  N
WT': GGATTTTTTTTTTAAATAGATTTAGGACCAATAAGCCTTAACTGGTTTGAAGAACTTTCTTCAGAAGCTCCACCCTATAAT
                                      L  G  P  I  S  L  N  W  F  E  E  L  S  S  E  A  P  P  Y  N
```

N30D – ssODN pair #2

```
Ref: GGATTTTTTTTTTAAATAGATTTAGGACCAATAAGTCTTAATTGGTTTGAAGAACTTTCTTCAGAAGCTCCACCCTATAAT
                                      L  G  P  I  S  L  N  W  F  E  E  L  S  S  E  A  P  P  Y  N
MUT: GGATTTTTTTTTTAAATAGATTTAGGACCAATAAGTCTTGATTGGTTTGAAGAACTTTCTTCAGAAGCTCCACCCTATAAT
                                      L  G  P  I  S  L  D  W  F  E  E  L  S  S  E  A  P  P  Y  N
WT': GGATTTTTTTTTTAAATAGATTTAGGACCAATAAGTCTTAACTGGTTTGAAGAACTTTCTTCAGAAGCTCCACCCTATAAT
                                      L  G  P  I  S  L  N  W  F  E  E  L  S  S  E  A  P  P  Y  N
```

N30H – ssODN pair #1

```
Ref: GGATTTTTTTTTTAAATAGATTTAGGACCAATAAGTCTTAATTGGTTTGAAGAACTTTCTTCAGAAGCTCCACCCTATAAT
                                      L  G  P  I  S  L  N  W  F  E  E  L  S  S  E  A  P  P  Y  N
MUT: GGATTTTTTTTTTAAATAGATTTAGGACCAATAAGCCTTCATTGGTTTGAAGAACTTTCTTCAGAAGCTCCACCCTATAAT
                                      L  G  P  I  S  L  H  W  F  E  E  L  S  S  E  A  P  P  Y  N
WT': GGATTTTTTTTTTAAATAGATTTAGGACCAATAAGCCTTAACTGGTTTGAAGAACTTTCTTCAGAAGCTCCACCCTATAAT
                                      L  G  P  I  S  L  N  W  F  E  E  L  S  S  E  A  P  P  Y  N
```

N30H – ssODN pair #2

```
Ref: GGATTTTTTTTTTAAATAGATTTAGGACCAATAAGTCTTAATTGGTTTGAAGAACTTTCTTCAGAAGCTCCACCCTATAAT
                                      L  G  P  I  S  L  N  W  F  E  E  L  S  S  E  A  P  P  Y  N
MUT: GGATTTTTTTTTTAAATAGATTTAGGACCAATAAGTCTTCATTGGTTTGAAGAACTTTCTTCAGAAGCTCCACCCTATAAT
                                      L  G  P  I  S  L  H  W  F  E  E  L  S  S  E  A  P  P  Y  N
WT': GGATTTTTTTTTTAAATAGATTTAGGACCAATAAGTCTTAACTGGTTTGAAGAACTTTCTTCAGAAGCTCCACCCTATAAT
                                      L  G  P  I  S  L  N  W  F  E  E  L  S  S  E  A  P  P  Y  N
```

N30S – ssODN pair #1

```
Ref: GGATTTTTTTTTTAAATAGATTTAGGACCAATAAGTCTTAATTGGTTTGAAGAACTTTCTTCAGAAGCTCCACCCTATAAT
                                      L  G  P  I  S  L  N  W  F  E  E  L  S  S  E  A  P  P  Y  N
MUT: GGATTTTTTTTTTAAATAGATTTAGGACCAATAAGCCTTAGTTGGTTTGAAGAACTTTCTTCAGAAGCTCCACCCTATAAT
                                      L  G  P  I  S  L  S  W  F  E  E  L  S  S  E  A  P  P  Y  N
WT': GGATTTTTTTTTTAAATAGATTTAGGACCAATAAGCCTTAACTGGTTTGAAGAACTTTCTTCAGAAGCTCCACCCTATAAT
                                      L  G  P  I  S  L  N  W  F  E  E  L  S  S  E  A  P  P  Y  N
```

N30S – ssODN pair #2

```
Ref: GGATTTTTTTTTTAAATAGATTTAGGACCAATAAGTCTTAATTGGTTTGAAGAACTTTCTTCAGAAGCTCCACCCTATAAT
                                      L  G  P  I  S  L  N  W  F  E  E  L  S  S  E  A  P  P  Y  N
MUT: GGATTTTTTTTTTAAATAGATTTAGGACCAATAAGTCTTAGTTGGTTTGAAGAACTTTCTTCAGAAGCTCCACCCTATAAT
                                      L  G  P  I  S  L  S  W  F  E  E  L  S  S  E  A  P  P  Y  N
WT': GGATTTTTTTTTTAAATAGATTTAGGACCAATAAGTCTTAACTGGTTTGAAGAACTTTCTTCAGAAGCTCCACCCTATAAT
                                      L  G  P  I  S  L  N  W  F  E  E  L  S  S  E  A  P  P  Y  N
```

N30K – ssODN pair #1

```
Ref: GGATTTTTTTTTTAAATAGATTTAGGACCAATAAGTCTTAATTGGTTTGAAGAACTTTCTTCAGAAGCTCCACCCTATAAT
     L G P I S L N W F E E L S S E A P P Y N
MUT: GGATTTTTTTTTTAAATAGATTTAGGACCAATAAGCCTTAAGTGGTTTGAAGAACTTTCTTCAGAAGCTCCACCCTATAAT
     L G P I S L K W F E E L S S E A P P Y N
WT': GGATTTTTTTTTTAAATAGATTTAGGACCAATAAGCCTTAACTGGTTTGAAGAACTTTCTTCAGAAGCTCCACCCTATAAT
     L G P I S L N W F E E L S S E A P P Y N
```

N30K – ssODN pair #2

```
Ref: GGATTTTTTTTTTAAATAGATTTAGGACCAATAAGTCTTAATTGGTTTGAAGAACTTTCTTCAGAAGCTCCACCCTATAAT
     L G P I S L N W F E E L S S E A P P Y N
MUT: GGATTTTTTTTTTAAATAGATTTAGGACCAATAAGTCTTAAGTGGTTTGAAGAACTTTCTTCAGAAGCTCCACCCTATAAT
     L G P I S L K W F E E L S S E A P P Y N
WT': GGATTTTTTTTTTAAATAGATTTAGGACCAATAAGTCTTAACTGGTTTGAAGAACTTTCTTCAGAAGCTCCACCCTATAAT
     L G P I S L N W F E E L S S E A P P Y N
```

W31R

```
Ref: TTTTTTTTTTAAATAGATTTAGGACCAATAAGTCTTAATTGGTTTGAAGAACTTTCTTCAGAAGCTCCACCCTATAATTCT
     L G P I S L N W F E E L S S E A P P Y N S
MUT: TTTTTTTTTTAAATAGATTTAGGACCAATAAGCCTGAATCGGTTTGAAGAACTTTCTTCAGAAGCTCCACCCTATAATTCT
     L G P I S L N R F E E L S S E A P P Y N S
WT': TTTTTTTTTTAAATAGATTTAGGACCAATAAGCCTGAATTGGTTTGAAGAACTTTCTTCAGAAGCTCCACCCTATAATTCT
     L G P I S L N W F E E L S S E A P P Y N S
```

W31G

```
Ref: TTTTTTTTTTAAATAGATTTAGGACCAATAAGTCTTAATTGGTTTGAAGAACTTTCTTCAGAAGCTCCACCCTATAATTCT
     L G P I S L N W F E E L S S E A P P Y N S
MUT: TTTTTTTTTTAAATAGATTTAGGACCAATAAGCCTGAATGGGTTTGAAGAACTTTCTTCAGAAGCTCCACCCTATAATTCT
     L G P I S L N G F E E L S S E A P P Y N S
WT': TTTTTTTTTTAAATAGATTTAGGACCAATAAGCCTGAATTGGTTTGAAGAACTTTCTTCAGAAGCTCCACCCTATAATTCT
     L G P I S L N W F E E L S S E A P P Y N S
```

W31L – ssODN pair #1

```
Ref: TTTTTTTTTTAAATAGATTTAGGACCAATAAGTCTTAATTGGTTTGAAGAACTTTCTTCAGAAGCTCCACCCTATAATTCT
     L G P I S L N W F E E L S S E A P P Y N S
MUT: TTTTTTTTTTAAATAGATTTAGGACCAATAAGCCTGAATTTGTTTGAAGAACTTTCTTCAGAAGCTCCACCCTATAATTCT
     L G P I S L N L F E E L S S E A P P Y N S
WT': TTTTTTTTTTAAATAGATTTAGGACCAATAAGCCTGAATTGGTTTGAAGAACTTTCTTCAGAAGCTCCACCCTATAATTCT
     L G P I S L N W F E E L S S E A P P Y N S
```

W31L – ssODN pair #2

```
Ref: TTTTTTTTTTAAATAGATTTAGGACCAATAAGTCTTAATTGGTTTGAAGAACTTTCTTCAGAAGCTCCACCCTATAATTCT
     L G P I S L N W F E E L S S E A P P Y N S
MUT: TTTTTTTTTTAAATAGATTTAGGACCAATAAGTCTTAATTTGTTTGAAGAACTTTCTTCAGAAGCTCCACCCTATAATTCT
     L G P I S L N L F E E L S S E A P P Y N S
WT': TTTTTTTTTTAAATAGATTTAGGACCAATAAGTCTTAACTGGTTTGAAGAACTTTCTTCAGAAGCTCCACCCTATAATTCT
     L G P I S L N W F E E L S S E A P P Y N S
```

W31C

```
Ref: TTTTTTTTTTAAATAGATTTAGGACCAATAAGTCTTAATTGGTTTGAAGAACTTTCTTCAGAAGCTCCACCCTATAATTCT
```

```
                      L G P I S L N W F E E L S S E A P P Y N S
MUT: TTTTTTTTTTAAATAGATTTAGGACCAATAAGTCTTAATTGCTTTGAAGAACTTTCTTCAGAAGCTCCACCCTATAATTCT
                      L G P I S L N C F E E L S S E A P P Y N S
WT': TTTTTTTTTTAAATAGATTTAGGACCAATAAGTCTTAACTGGTTTGAAGAACTTTCTTCAGAAGCTCCACCCTATAATTCT
                      L G P I S L N W F E E L S S E A P P Y N S
```

F32L (c.94T>C)

```
Ref: TTTTTTTAAATAGATTTAGGACCAATAAGTCTTAATTGGTTTGAAGAACTTTCTTCAGAAGCTCCACCCTATAATTCTGAA
                    L G P I S L N W F E E L S S E A P P Y N S E
MUT: TTTTTTTAAATAGATTTAGGACCAATAAGCCTGAATTGGCTTGAAGAACTTTCTTCAGAAGCTCCACCCTATAATTCTGAA
                    L G P I S L N W L E E L S S E A P P Y N S E
WT': TTTTTTTAAATAGATTTAGGACCAATAAGCCTGAATTGGTTCGAAGAACTTTCTTCAGAAGCTCCACCCTATAATTCTGAA
                    L G P I S L N W F E E L S S E A P P Y N S E
```

F32L (c.96T>G)

```
Ref: TTTTTTTAAATAGATTTAGGACCAATAAGTCTTAATTGGTTTGAAGAACTTTCTTCAGAAGCTCCACCCTATAATTCTGAA
                    L G P I S L N W F E E L S S E A P P Y N S E
MUT: TTTTTTTAAATAGATTTAGGACCAATAAGCCTGAATTGGTTGGAAGAACTTTCTTCAGAAGCTCCACCCTATAATTCTGAA
                    L G P I S L N W L E E L S S E A P P Y N S E
WT': TTTTTTTAAATAGATTTAGGACCAATAAGCCTGAATTGGTTCGAAGAACTTTCTTCAGAAGCTCCACCCTATAATTCTGAA
                    L G P I S L N W F E E L S S E A P P Y N S E
```

E33*

```
Ref: TTTTAAATAGATTTAGGACCAATAAGTCTTAATTGGTTTGAAGAACTTTCTTCAGAAGCTCCACCCTATAATTCTGAACCT
                  L G P I S L N W F E E L S S E A P P Y N S E P
MUT: TTTTAAATAGATTTAGGACCAATAAGCCTGAATTGGTTTTAAGAACTTTCTTCAGAAGCTCCACCCTATAATTCTGAACCT
                  L G P I S L N W F - E L S S E A P P Y N S E P
WT': TTTTAAATAGATTTAGGACCAATAAGCCTGAATTGGTTTGAGGAACTTTCTTCAGAAGCTCCACCCTATAATTCTGAACCT
                  L G P I S L N W F E E L S S E A P P Y N S E P
```

E33K

```
Ref: TTTTAAATAGATTTAGGACCAATAAGTCTTAATTGGTTTGAAGAACTTTCTTCAGAAGCTCCACCCTATAATTCTGAACCT
                  L G P I S L N W F E E L S S E A P P Y N S E P
MUT: TTTTAAATAGATTTAGGACCAATAAGCCTGAATTGGTTTAAAGAACTTTCTTCAGAAGCTCCACCCTATAATTCTGAACCT
                  L G P I S L N W F K E L S S E A P P Y N S E P
WT': TTTTAAATAGATTTAGGACCAATAAGCCTGAATTGGTTTGAGGAACTTTCTTCAGAAGCTCCACCCTATAATTCTGAACCT
                  L G P I S L N W F E E L S S E A P P Y N S E P
```

L35F

```
Ref: ATAGATTTAGGACCAATAAGTCTTAATTGGTTTGAAGAACTTTCTTCAGAAGCTCCACCCTATAATTCTGAACCTGCAGAA
              L G P I S L N W F E E L S S E A P P Y N S E P A E
MUT: ATAGATTTAGGACCAATAAGCCTGAATTGGTTTGAAGAATTTTCTTCAGAAGCTCCACCCTATAATTCTGAACCTGCAGAA
              L G P I S L N W F E E F S S E A P P Y N S E P A E
WT': ATAGATTTAGGACCAATAAGCCTGAATTGGTTTGAAGAACTGTCTTCAGAAGCTCCACCCTATAATTCTGAACCTGCAGAA
              L G P I S L N W F E E L S S E A P P Y N S E P A E
```

S36Y

```
Ref: GATTTAGGACCAATAAGTCTTAATTGGTTTGAAGAACTTTCTTCAGAAGCTCCACCCTATAATTCTGAACCTGCAGAAGAA
            L G P I S L N W F E E L S S E A P P Y N S E P A E E
MUT: GATTTAGGACCAATAAGTCTTAATTGGTTTGAAGAACTTTATTCAGAAGCTCCACCATACAATTCTGAACCTGCAGAAGAA
            L G P I S L N W F E E L Y S E A P P Y N S E P A E E
```

```
WT': GATTTAGGACCAATAAGTCTTAATTGGTTTGAAGAACTTTCATCAGAAGCTCCACCATACAATTCTGAACCTGCAGAAGAA
        L  G  P  I  S  L  N  W  F  E  E  L  S  S  E  A  P  P  Y  N  S  E  P  A  E  E
```

S36F

```
Ref: GATTTAGGACCAATAAGTCTTAATTGGTTTGAAGAACTTTCTTCAGAAGCTCCACCCTATAATTCTGAACCTGCAGAAGAA
        L  G  P  I  S  L  N  W  F  E  E  L  S  S  E  A  P  P  Y  N  S  E  P  A  E  E
MUT: GATTTAGGACCAATAAGTCTTAATTGGTTTGAAGAACTTTTTTCAGAAGCTCCACCATACAATTCTGAACCTGCAGAAGAA
        L  G  P  I  S  L  N  W  F  E  E  L  F  S  E  A  P  P  Y  N  S  E  P  A  E  E
WT': GATTTAGGACCAATAAGTCTTAATTGGTTTGAAGAACTTTCATCAGAAGCTCCACCATACAATTCTGAACCTGCAGAAGAA
        L  G  P  I  S  L  N  W  F  E  E  L  S  S  E  A  P  P  Y  N  S  E  P  A  E  E
```

S37A

```
Ref: TTAGGACCAATAAGTCTTAATTGGTTTGAAGAACTTTCTTCAGAAGCTCCACCCTATAATTCTGAACCTGCAGAAGAATCT
      L  G  P  I  S  L  N  W  F  E  E  L  S  S  E  A  P  P  Y  N  S  E  P  A  E  E  S
MUT: TTAGGACCAATAAGTCTTAATTGGTTTGAAGAACTTTCTGCAGAAGCTCCACCATACAATTCTGAACCTGCAGAAGAATCT
      L  G  P  I  S  L  N  W  F  E  E  L  S  A  E  A  P  P  Y  N  S  E  P  A  E  E  S
WT': TTAGGACCAATAAGTCTTAATTGGTTTGAAGAACTTTCTTCTGAAGCTCCACCATACAATTCTGAACCTGCAGAAGAATCT
      L  G  P  I  S  L  N  W  F  E  E  L  S  S  E  A  P  P  Y  N  S  E  P  A  E  E  S
```

S37L

```
Ref: TTAGGACCAATAAGTCTTAATTGGTTTGAAGAACTTTCTTCAGAAGCTCCACCCTATAATTCTGAACCTGCAGAAGAATCT
      L  G  P  I  S  L  N  W  F  E  E  L  S  S  E  A  P  P  Y  N  S  E  P  A  E  E  S
MUT: TTAGGACCAATAAGTCTTAATTGGTTTGAAGAACTTTCTTTAGAAGCTCCACCATACAATTCTGAACCTGCAGAAGAATCT
      L  G  P  I  S  L  N  W  F  E  E  L  S  L  E  A  P  P  Y  N  S  E  P  A  E  E  S
WT': TTAGGACCAATAAGTCTTAATTGGTTTGAAGAACTTTCTTCTGAAGCTCCACCATACAATTCTGAACCTGCAGAAGAATCT
      L  G  P  I  S  L  N  W  F  E  E  L  S  S  E  A  P  P  Y  N  S  E  P  A  E  E  S
```

S37*

```
Ref: TTAGGACCAATAAGTCTTAATTGGTTTGAAGAACTTTCTTCAGAAGCTCCACCCTATAATTCTGAACCTGCAGAAGAATCT
      L  G  P  I  S  L  N  W  F  E  E  L  S  S  E  A  P  P  Y  N  S  E  P  A  E  E  S
MUT: TTAGGACCAATAAGTCTTAATTGGTTTGAAGAACTTTCTTGAGAAGCTCCACCATACAATTCTGAACCTGCAGAAGAATCT
      L  G  P  I  S  L  N  W  F  E  E  L  S  -  E  A  P  P  Y  N  S  E  P  A  E  E  S
WT': TTAGGACCAATAAGTCTTAATTGGTTTGAAGAACTTTCTTCTGAAGCTCCACCATACAATTCTGAACCTGCAGAAGAATCT
      L  G  P  I  S  L  N  W  F  E  E  L  S  S  E  A  P  P  Y  N  S  E  P  A  E  E  S
```

E38K

```
Ref: GGACCAATAAGTCTTAATTGGTTTGAAGAACTTTCTTCAGAAGCTCCACCCTATAATTCTGAACCTGCAGAAGAATCTGAA
      G  P  I  S  L  N  W  F  E  E  L  S  S  E  A  P  P  Y  N  S  E  P  A  E  E  S  E
MUT: GGACCAATAAGTCTTAATTGGTTTGAAGAACTTTCTTCAAAAGCTCCACCATACAATTCTGAACCTGCAGAAGAATCTGAA
      G  P  I  S  L  N  W  F  E  E  L  S  S  K  A  P  P  Y  N  S  E  P  A  E  E  S  E
WT': GGACCAATAAGTCTTAATTGGTTTGAAGAACTTTCTTCAGAGGCTCCACCATACAATTCTGAACCTGCAGAAGAATCTGAA
      G  P  I  S  L  N  W  F  E  E  L  S  S  E  A  P  P  Y  N  S  E  P  A  E  E  S  E
```

A39=

```
Ref: CCAATAAGTCTTAATTGGTTTGAAGAACTTTCTTCAGAAGCTCCACCCTATAATTCTGAACCTGCAGAAGAATCTGAACAT
      P  I  S  L  N  W  F  E  E  L  S  S  E  A  P  P  Y  N  S  E  P  A  E  E  S  E  H
MUT: CCAATAAGTCTTAATTGGTTTGAAGAACTTTCTTCAGAAGCCCCACCATACAATTCTGAACCTGCAGAAGAATCTGAACAT
      P  I  S  L  N  W  F  E  E  L  S  S  E  A  P  P  Y  N  S  E  P  A  E  E  S  E  H
WT': CCAATAAGTCTTAATTGGTTTGAAGAACTTTCTTCAGAAGCACCACCATACAATTCTGAACCTGCAGAAGAATCTGAACAT
      P  I  S  L  N  W  F  E  E  L  S  S  E  A  P  P  Y  N  S  E  P  A  E  E  S  E  H
```

H2623Y

Ref: CAGGTGTGGATCCAAAGCTTATTTCTAGAATTTGGGTTTATAATCACTATAGA**TGG**ATCATATGGAAACTGGCAGCTATGGAATGTGCCT
G V D P K L I S R I W V Y N H Y R W I I W K L A A M E C A
MUT: CAGGTGTGGATCCAAAGCTTATTTCTAGAATTTGGGTTTATAATTACTATAGA**TGG**ATCATATGGAAACTGGCAGCTATGGAATGTGCCT
G V D P K L I S R I W V Y N **Y** Y R W I I W K L A A M E C A
WT′: CAGGTGTGGATCCAAAGCTTATTTCTAGAATTTGGGTTTATAATCATTATAGA**TGG**ATCATATGGAAACTGGCAGCTATGGAATGTGCCT
G V D P K L I S R I W V Y N H Y R W I I W K L A A M E C A

H2623R

Ref: CAGGTGTGGATCCAAAGCTTATTTCTAGAATTTGGGTTTATAATCACTATAGA**TGG**ATCATATGGAAACTGGCAGCTATGGAATGTGCCT
G V D P K L I S R I W V Y N H Y R W I I W K L A A M E C A
MUT: CAGGTGTGGATCCAAAGCTTATTTCTAGAATTTGGGTTTATAATCGCTATAGA**TGG**ATCATATGGAAACTGGCAGCTATGGAATGTGCCT
G V D P K L I S R I W V Y N **R** Y R W I I W K L A A M E C A
WT′: CAGGTGTGGATCCAAAGCTTATTTCTAGAATTTGGGTTTATAATCATTATAGA**TGG**ATCATATGGAAACTGGCAGCTATGGAATGTGCCT
G V D P K L I S R I W V Y N H Y R W I I W K L A A M E C A

W2626C

Ref: TCCAAAGCTTATTTCTAGAATTTGGGTTTATAATCACTATAGATGGATCATA**TGG**AAACTGGCAGCTATGGAATGTGCCTTTCCTAAGGA
P K L I S R I W V Y N H Y R W I I W K L A A M E C A F P K
MUT: TCCAAAGCTTATTTCTAGAATTTGGGTTTATAATCACTATAGATGCATCATA**TGG**AAACTGGCAGCTATGGAATGTGCCTTTCCTAAGGA
P K L I S R I W V Y N H Y R **C** I I W K L A A M E C A F P K
WT′: TCCAAAGCTTATTTCTAGAATTTGGGTTTATAATCACTATAGATGGATAATA**TGG**AAACTGGCAGCTATGGAATGTGCCTTTCCTAAGGA
P K L I S R I W V Y N H Y R W I I W K L A A M E C A F P K

I2627F

Ref: TCCAAAGCTTATTTCTAGAATTTGGGTTTATAATCACTATAGATGGATCATA**TGG**AAACTGGCAGCTATGGAATGTGCCTTTCCTAAGGA
P K L I S R I W V Y N H Y R W I I W K L A A M E C A F P K
MUT: TCCAAAGCTTATTTCTAGAATTTGGGTTTATAATCACTATAGATGGTTCATA**TGG**AAACTGGCAGCTATGGAATGTGCCTTTCCTAAGGA
P K L I S R I W V Y N H Y R W **F** I W K L A A M E C A F P K
WT′: TCCAAAGCTTATTTCTAGAATTTGGGTTTATAATCACTATAGATGGATAATA**TGG**AAACTGGCAGCTATGGAATGTGCCTTTCCTAAGGA
P K L I S R I W V Y N H Y R W I I W K L A A M E C A F P K

T2722R

Ref: AAACTAGTAGTGCAGATACCCAAAAAGTGGCCATTATTGAACTTACAGATGGG**TGG**TATGCTGTTAAGGCCCAGTTAGATCCTCCCCTCT
T S S A D T Q K V A I I E L T D G W Y A V K A Q L D P P L
MUT: AAACTAGTAGTGCAGATACCCAAAAAGTGGCCATTATTGAACTTAGAGATGGG**TGG**TATGCTGTTAAGGCCCAGTTAGATCCTCCCCTCT
T S S A D T Q K V A I I E L **R** D G W Y A V K A Q L D P P L
WT′: AAACTAGTAGTGCAGATACCCAAAAAGTGGCCATTATTGAACTTACTGATGGG**TGG**TATGCTGTTAAGGCCCAGTTAGATCCTCCCCTCT
T S S A D T Q K V A I I E L T D G W Y A V K A Q L D P P L

D2723A

Ref: AAACTAGTAGTGCAGATACCCAAAAAGTGGCCATTATTGAACTTACAGATGGG**TGG**TATGCTGTTAAGGCCCAGTTAGATCCTCCCCTCTTA
T S S A D T Q K V A I I E L T D G W Y A V K A Q L D P P L L
MUT: AAACTAGTAGTGCAGATACCCAAAAAGTGGCCATTATTGAACTTACAGCTGGG**TGG**TATGCTGTTAAGGCCCAGTTAGATCCTCCCCTCTTA
T S S A D T Q K V A I I E L T **A** G W Y A V K A Q L D P P L L
WT′: AAACTAGTAGTGCAGATACCCAAAAAGTGGCCATTATTGAACTTACAGACGGG**TGG**TATGCTGTTAAGGCCCAGTTAGATCCTCCCCTCTTA
T S S A D T Q K V A I I E L T D G W Y A V K A Q L D P P L L

D2723G

Ref: AAACTAGTAGTGCAGATACCCAAAAAGTGGCCATTATTGAACTTACAGATGGG**TGG**TATGCTGTTAAGGCCCAGTTAGATCCTCCCCTCTTA

```
        T  S  S  A  D  T  Q  K  V  A  I  I  E  L  T  D  G  W  Y  A  V  K  A  Q  L  D  P  P  L  L
MUT:  AAACTAGTAGTGCAGATACCCAAAAAGTGGCCATTATTGAACTTACAGGTGGGTGGTATGCTGTTAAGGCCCAGTTAGATCCTCCCCTCTTA
        T  S  S  A  D  T  Q  K  V  A  I  I  E  L  T  G  G  W  Y  A  V  K  A  Q  L  D  P  P  L  L
WT':  AAACTAGTAGTGCAGATACCCAAAAAGTGGCCATTATTGAACTTACAGACGGGTGGTATGCTGTTAAGGCCCAGTTAGATCCTCCCCTCTTA
        T  S  S  A  D  T  Q  K  V  A  I  I  E  L  T  D  G  W  Y  A  V  K  A  Q  L  D  P  P  L  L
```

V2728I

```
Ref:  CCCAAAAAGTGGCCATTATTGAACTTACAGATGGGTGGTATGCTGTTAAGGCCCAGTTAGATCCTCCCCTCTTAGCTGTCTTAAAGAATG
        Q  K  V  A  I  I  E  L  T  D  G  W  Y  A  V  K  A  Q  L  D  P  P  L  L  A  V  L  K  N
MUT:  CCCAAAAAGTGGCCATTATTGAACTTACAGATGGGTGGTATGCTATTAAGGCCCAGTTAGATCCTCCCCTCTTAGCTGTCTTAAAGAATG
        Q  K  V  A  I  I  E  L  T  D  G  W  Y  A  I  K  A  Q  L  D  P  P  L  L  A  V  L  K  N
WT':  CCCAAAAAGTGGCCATTATTGAACTTACAGATGGGTGGTATGCTGTAAAGGCCCAGTTAGATCCTCCCCTCTTAGCTGTCTTAAAGAATG
        Q  K  V  A  I  I  E  L  T  D  G  W  Y  A  V  K  A  Q  L  D  P  P  L  L  A  V  L  K  N
```

K2729N

```
Ref:  AAAAAGTGGCCATTATTGAACTTACAGATGGGTGGTATGCTGTTAAGGCCCAGTTAGATCCTCCCCTCTTAGCTGTCTTAAAGAATGGCA
        K  V  A  I  I  E  L  T  D  G  W  Y  A  V  K  A  Q  L  D  P  P  L  L  A  V  L  K  N  G
MUT:  AAAAAGTGGCCATTATTGAACTTACAGATGGGTGGTATGCTGTTAATGCCCAGTTAGATCCTCCCCTCTTAGCTGTCTTAAAGAATGGCA
        K  V  A  I  I  E  L  T  D  G  W  Y  A  V  N  A  Q  L  D  P  P  L  L  A  V  L  K  N  G
WT':  AAAAAGTGGCCATTATTGAACTTACAGATGGGTGGTATGCTGTTAAAGCCCAGTTAGATCCTCCCCTCTTAGCTGTCTTAAAGAATGGCA
        K  V  A  I  I  E  L  T  D  G  W  Y  A  V  K  A  Q  L  D  P  P  L  L  A  V  L  K  N  G
```

Y3035S

```
Ref:  CTGAAAGAGCTAACATACAGTTAGCAGCGACAAAAAAAAACTCAGTATCAACAACTACCGGTACAAACCTTTCATTGTAATTTTTCAGTTT
        E  R  A  N  I  Q  L  A  A  T  K  K  T  Q  Y  Q  Q  L  P
MUT:  CTGAAAGAGCTAACATACAGTTAGCAGCGACAAAAAAAAACTCAGTCTCAACAACTACCGGTACAAACCTTTCATTGTAATTTTTCAGTTT
        E  R  A  N  I  Q  L  A  A  T  K  K  T  Q  S  Q  Q  L  P
WT':  CTGAAAGAGCTAACATACAGTTAGCAGCGACAAAAAAAAACTCAGTACCAACAACTACCGGTACAAACCTTTCATTGTAATTTTTCAGTTT
        E  R  A  N  I  Q  L  A  A  T  K  K  T  Q  Y  Q  Q  L  P
```

L3074=

```
Ref:  TTTTTAGATCCAGACTTTCAGCCATCTTGTTCTGAGGTGGACCTAATAGGATTTGTCGTTTCTGTTGTGAAAAAAACAGGTAATGCACAA
       F  L  D  P  D  F  Q  P  S  C  S  E  V  D  L  I  G  F  V  V  S  V  V  K  K  T
MUT:  TTTTTAGATCCAGACTTTCAGCCATCTTGTTCTGAGGTGGACCTGATAGGATTTGTCGTTTCTGTTGTGAAAAAAACAGGTAATGCACAA
       F  L  D  P  D  F  Q  P  S  C  S  E  V  D  L  I  G  F  V  V  S  V  V  K  K  T
WT':  TTTTTAGATCCAGACTTTCAGCCATCTTGTTCTGAGGTGGACCTTATAGGATTTGTCGTTTCTGTTGTGAAAAAAACAGGTAATGCACAA
       F  L  D  P  D  F  Q  P  S  C  S  E  V  D  L  I  G  F  V  V  S  V  V  K  K  T
```

G3076V

```
Ref:  TAGATCCAGACTTTCAGCCATCTTGTTCTGAGGTGGACCTAATAGGATTTGTCGTTTCTGTTGTGAAAAAAACAGGTAATGCACAATATA
        D  P  D  F  Q  P  S  C  S  E  V  D  L  I  G  F  V  V  S  V  V  K  K  T
MUT:  TAGATCCAGACTTTCAGCCATCTTGTTCTGAGGTGGACCTAATAGTATTTGTCGTTTCTGTTGTGAAAAAAACAGGTAATGCACAATATA
        D  P  D  F  Q  P  S  C  S  E  V  D  L  I  V  F  V  V  S  V  V  K  K  T
WT':  TAGATCCAGACTTTCAGCCATCTTGTTCTGAGGTGGACCTAATAGGTTTTGTCGTTTCTGTTGTGAAAAAAACAGGTAATGCACAATATA
        D  P  D  F  Q  P  S  C  S  E  V  D  L  I  G  F  V  V  S  V  V  K  K  T
```

G3076E

```
Ref:  TAGATCCAGACTTTCAGCCATCTTGTTCTGAGGTGGACCTAATAGGATTTGTCGTTTCTGTTGTGAAAAAAACAGGTAATGCACAATATA
        D  P  D  F  Q  P  S  C  S  E  V  D  L  I  G  F  V  V  S  V  V  K  K  T
MUT:  TAGATCCAGACTTTCAGCCATCTTGTTCTGAGGTGGACCTAATAGAATTTGTCGTTTCTGTTGTGAAAAAAACAGGTAATGCACAATATA
        D  P  D  F  Q  P  S  C  S  E  V  D  L  I  E  F  V  V  S  V  V  K  K  T
```

WT′: TAGATCCAGACTTTCAGCCATCTTGTTCTGAGGTGGA**CCT**AATAGGTTTTGTCGTTTCTGTTGTGAAAAAAACAGGTAATGCACAATATA
D P D F Q P S C S E V D L I G F V V S V V K K T

V3079I

Ref: TTTCAGCCATCTTGTTCTGAGGTGGA**CCT**AATAGGATTTGTCGTTTCTGTTGTGAAAAAAACAGGTAATGCACAATATAGTTAATTTTTT
F Q P S C S E V D L I G F V V S V V K K T

MUT: TTTCAGCCATCTTGTTCTGAGGTGGA**CCT**AATAGGATTTGTCATTTCTGTTGTGAAAAAAACAGGTAATGCACAATATAGTTAATTTTTT
F Q P S C S E V D L I G F V **I** S V V K K T

WT′: TTTCAGCCATCTTGTTCTGAGGTGGA**CCT**AATAGGATTTGTCGTATCTGTTGTGAAAAAAACAGGTAATGCACAATATAGTTAATTTTTT
F Q P S C S E V D L I G F V V S V V K K T

Supplemental Table 7. List of all primers used in this study

***BRCA2* exon 2 variants:** T10P, T10I, T10K, T10=, F11L (c.31T>C), F11V, F11C, F11L (c.33T>G), F12S, E13*, E13K, E13V, E13D, I14V, I14N, I14M, F15L, F15C, K16R, T17I, T17=, R18H, C19R, C19Y, C19F, A22= (c.66A>C), A22= (c.66A>G), A22= (c.66A>T), D23H, were amplified using	Forward primer	ACACTCTTTCCCTACACGACGCTCTTCCGATCTATGCATCCCTGTGTAAGTGC
	Reverse primer	TGACTGGAGTTCAGACGTGTGCTCTTCCGATCTAGCAACACTGTGACGTACTG
***BRCA2* exon 3 variants:** D23G, D23V, L24V, L24F, G25*, G25R, G25E, P26S, P26R, P26L, P26=, S28G, S28N, L29V, L29R, N30D, N30H, N30S, N30K, W31R, W31G, W31L, W31C, F32L (c.94T>C), F32L (c.96T>G), E33*, E33K, L35F, S36Y, S36F, S37A, S37L, S37*, E38K, A39=, were amplified using	Forward primer	ACACTCTTTCCCTACACGACGCTCTTCCGATCTACTGTTCTGGGTCACAAATTTG
	Reverse primer	TGACTGGAGTTCAGACGTGTGCTCTTCCGATCTAGGTTTGGTTCGTAATTGTTGTT
***BRCA2* exon 17 variants:** H2623Y, H2623R, W2626C, I2627F, were amplified using	Forward primer	ACACTCTTTCCCTACACGACGCTCTTCCGATCTGGTGTGGATCCAAAGCTTATTTC
	Reverse primer	TGACTGGAGTTCAGACGTGTGCTCTTCCGATCTGAAACCTTAACCATACTGCCGT
***BRCA2* exon 18 variants:** T2722R, D2723A, D2723G, V2728I, K2729N, were amplified using	Forward primer	ACACTCTTTCCCTACACGACGCTCTTCCGATCTGATGACACAGCTGCAAAAACAC
	Reverse primer	TGACTGGAGTTCAGACGTGTGCTCTTCCGATCTGCTTCAAGAGGTGTACAGGCA

***BRCA2* exon 23 variant:** Y3035S was amplified using	Forward primer	ACACTCTTTCCCTACACGACGCTCTTCCGATCTACTGAGTATTTGGCGTCCATCA
	Reverse primer	TGACTGGAGTTCAGACGTGTGCTCTTCCGATCTTGGAGATTCCATAAACTAACAAGCAC
***BRCA2* exon 24 variants:** L3074=, G3076V, G3076E, V3079I, were amplified using	Forward primer	ACACTCTTTCCCTACACGACGCTCTTCCGATCTGCCCCTTCACTTCAGCAAAT
	Reverse primer	TGACTGGAGTTCAGACGTGTGCTCTTCCGATCTTCAGAGGTTCAAAGAGGCTTACT

Supplementary Figure 1

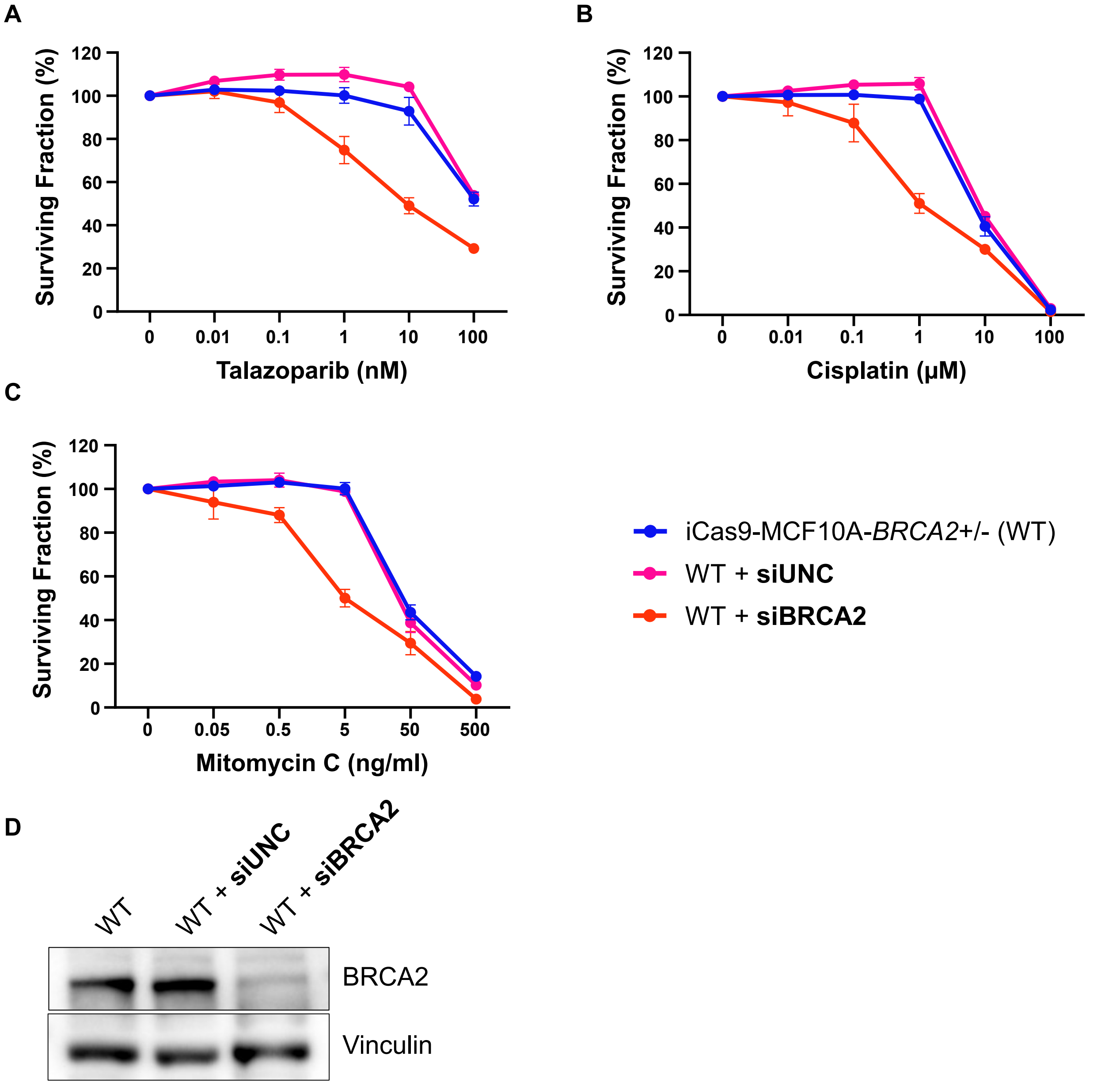

Supplementary Figure 2

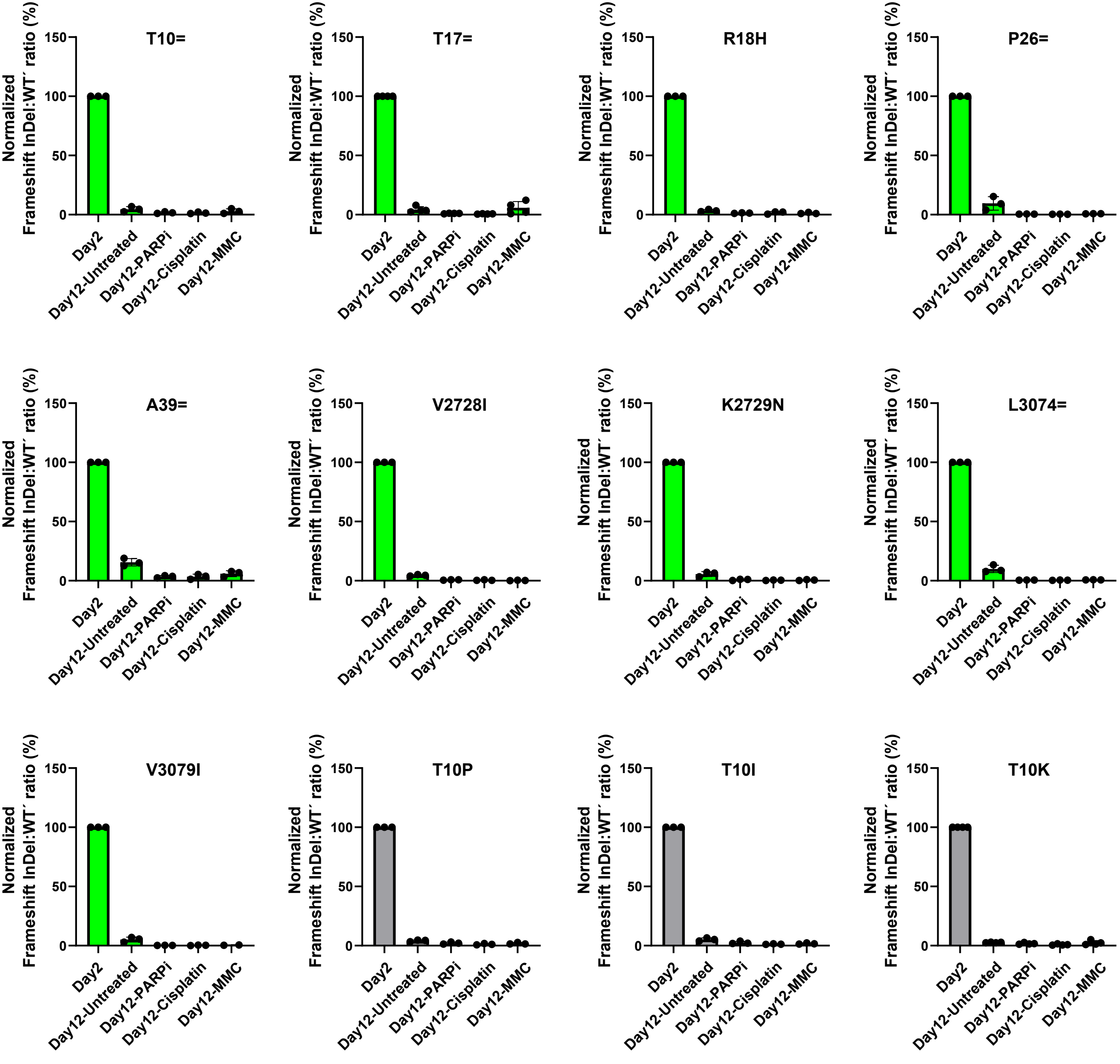

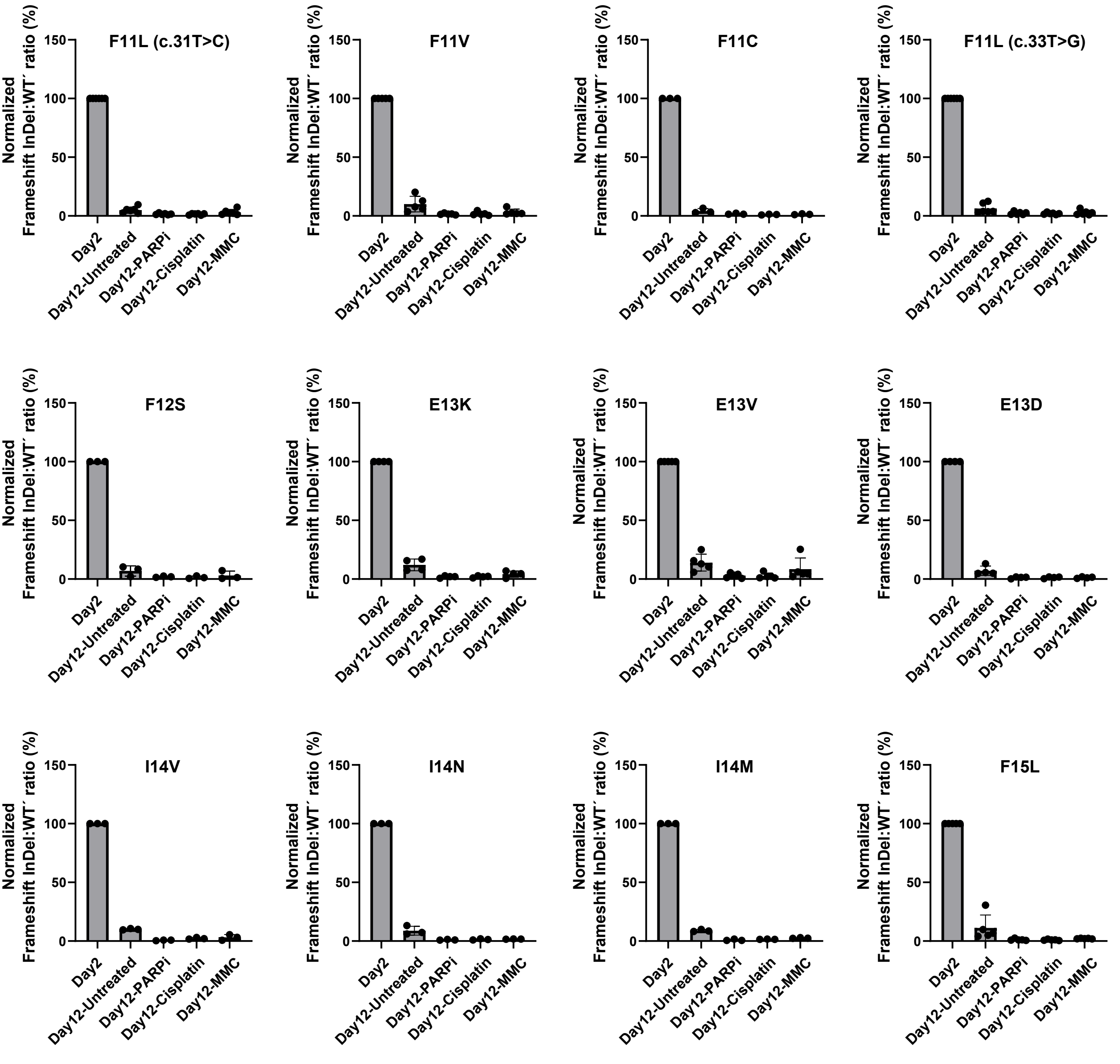

F11L (c.31T>C)
F11V
F11C
F11L (c.33T>G)
F12S
E13K
E13V
E13D
I14V
I14N
I14M
F15L
Normalized
Frameshift InDel:WT´ ratio (%)
150
100
50
0
Day2
Day12-Untreated
Day12-PARPi
Day12-Cisplatin
Day12-MMC

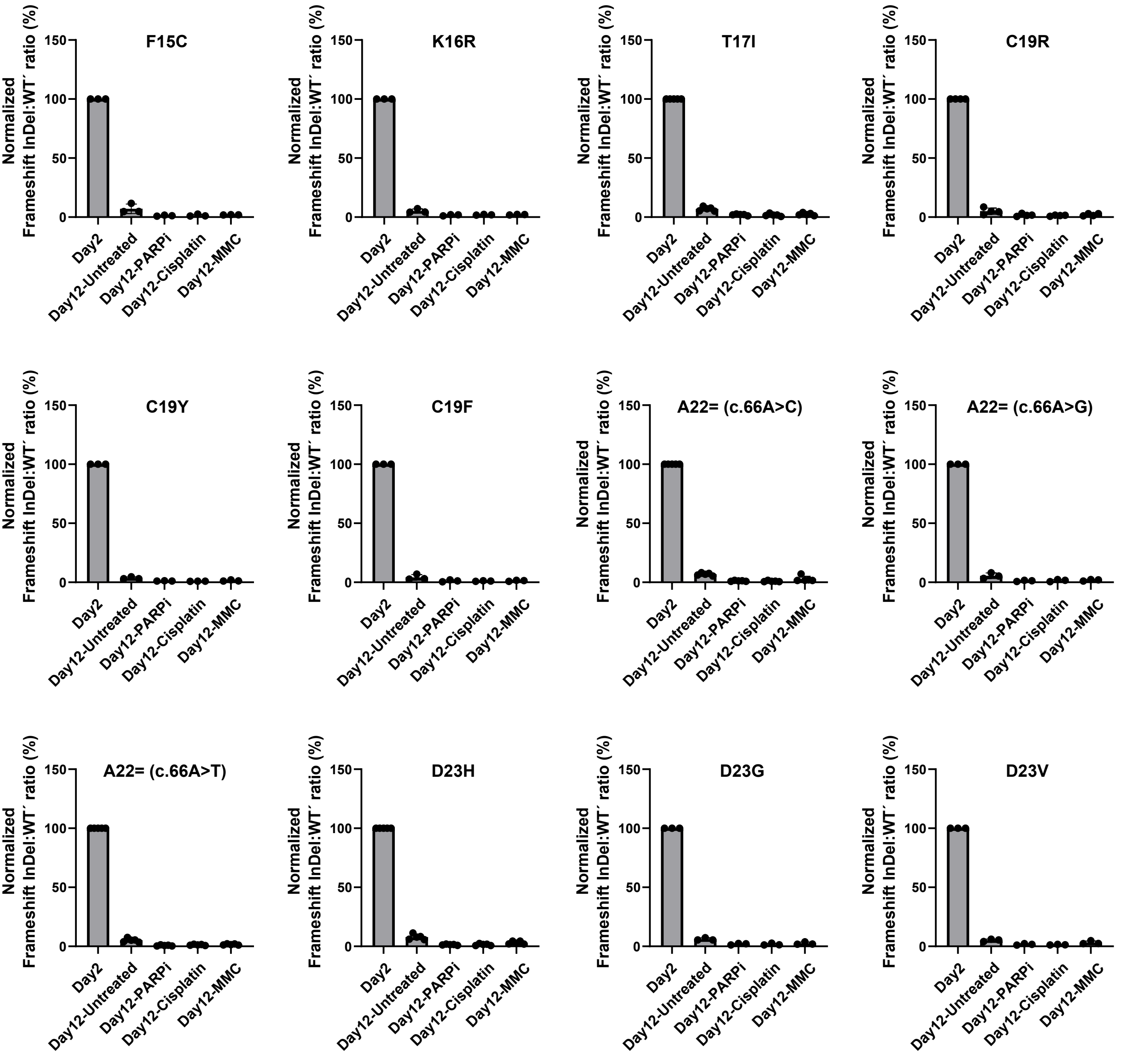
F15C
K16R
T17I
C19R
C19Y
C19F
A22= (c.66A>C)
A22= (c.66A>G)
A22= (c.66A>T)
D23H
D23G
D23V
Normalized
Frameshift InDel:WT´ ratio (%)
150
100
50
0
Day2
Day12-Untreated
Day12-PARPi
Day12-Cisplatin
Day12-MMC

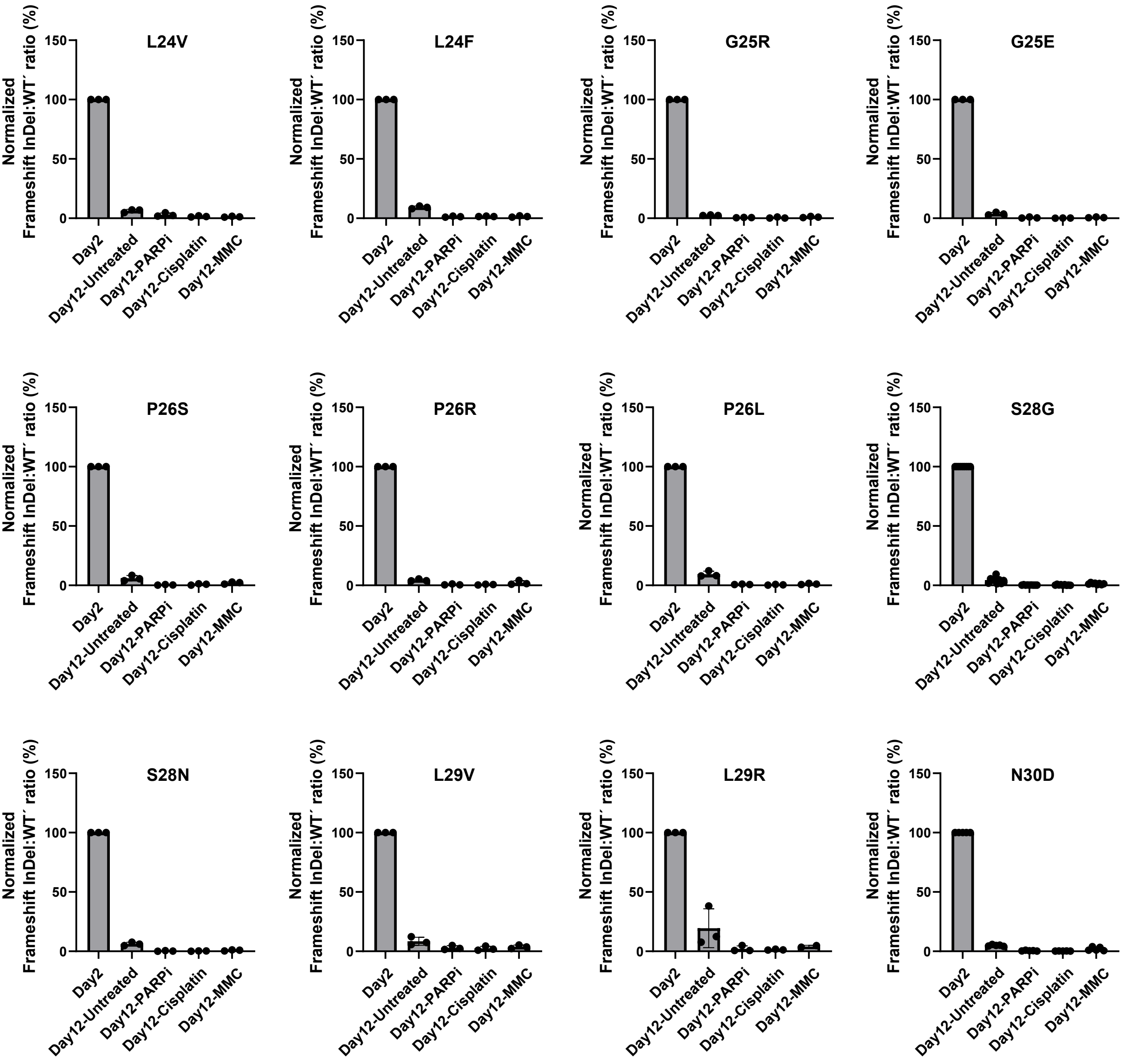

L24V
L24F
G25R
G25E
P26S
P26R
P26L
S28G
S28N
L29V
L29R
N30D
Normalized
Frameshift InDel:WT´ ratio (%)
150
100
50
0
Day2
Day12-Untreated
Day12-PARPi
Day12-Cisplatin
Day12-MMC

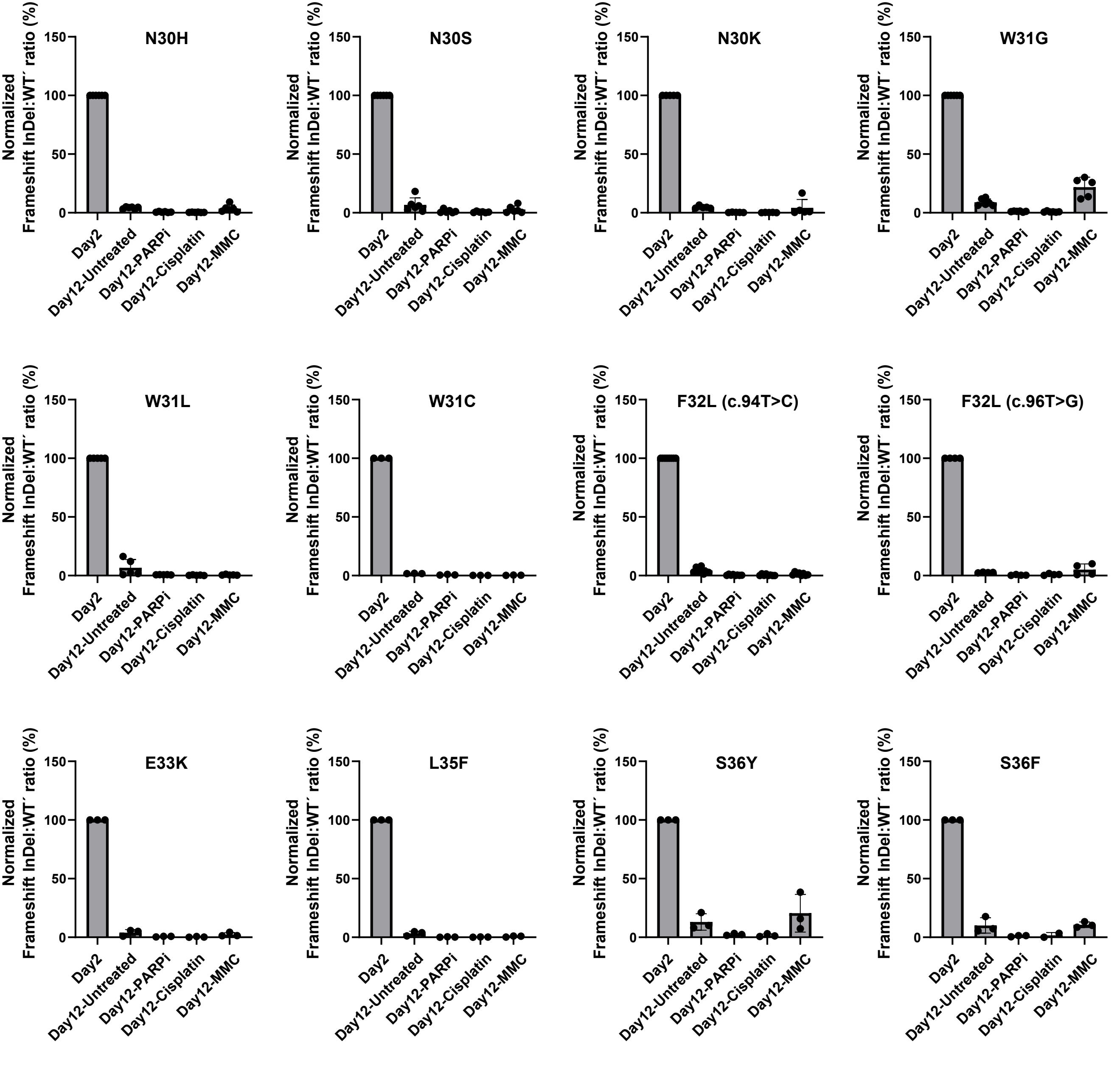
N30H
N30S
N30K
W31G
W31L
W31C
F32L (c.94T>C)
F32L (c.96T>G)
E33K
L35F
S36Y
S36F
Normalized
Frameshift InDel:WT´ ratio (%)
0
50
100
150
Day2
Day12-Untreated
Day12-PARPi
Day12-Cisplatin
Day12-MMC

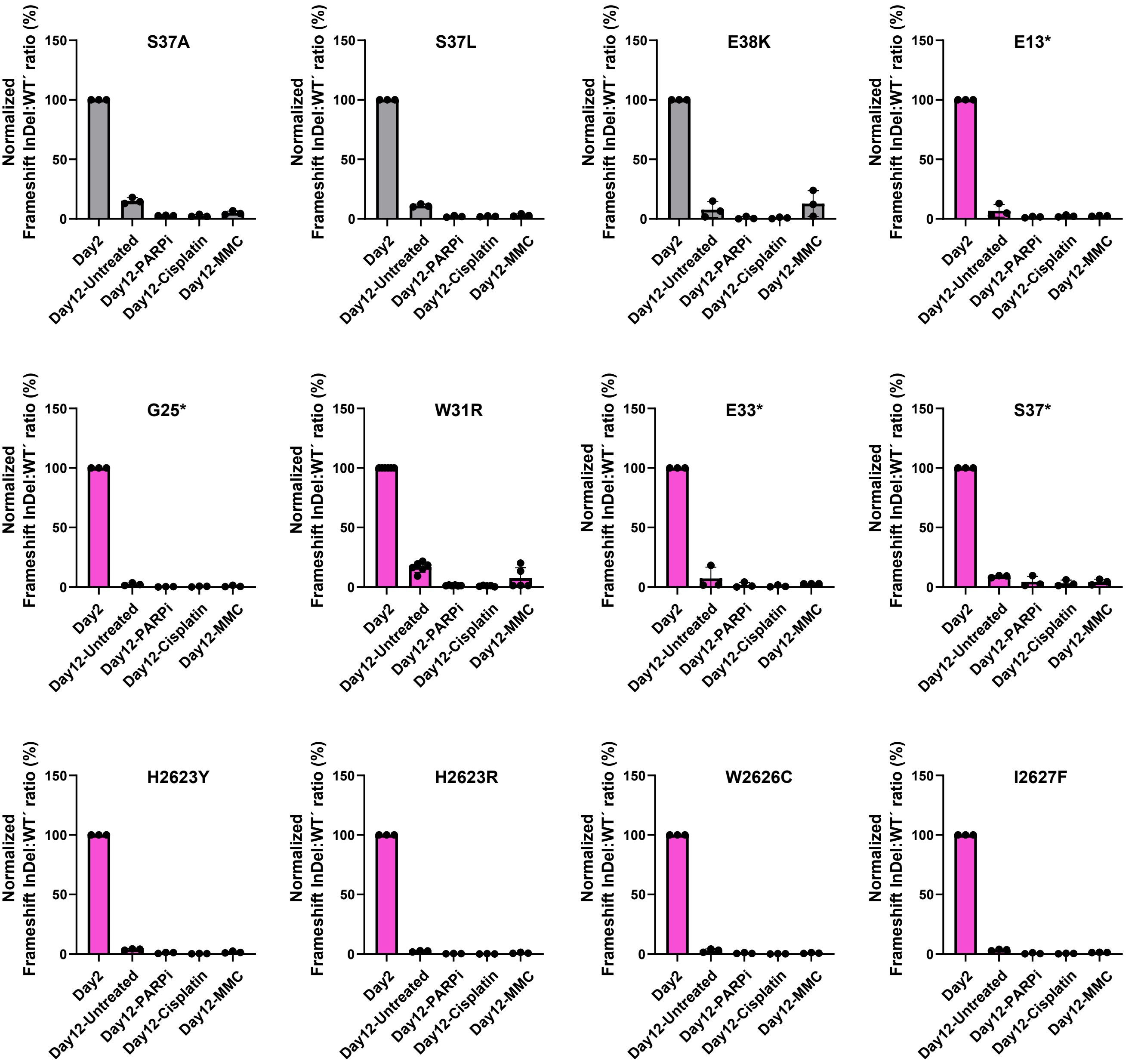

S37A
S37L
E38K
E13*
G25*
W31R
E33*
S37*
H2623Y
H2623R
W2626C
I2627F
Normalized
Frameshift InDel:WT´ ratio (%)
150
100
50
0
Day2
Day12-Untreated
Day12-PARPi
Day12-Cisplatin
Day12-MMC

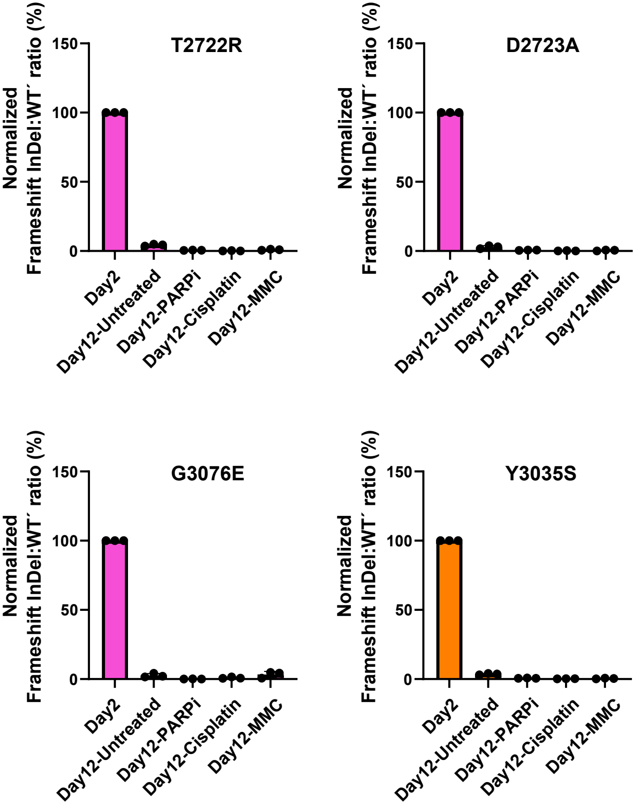
T2722R
D2723A
D2723G
G3076V
G3076E
Y3035S
Normalized Frameshift InDel:WT´ ratio (%)
150
100
50
0
Day2
Day12-Untreated
Day12-PARPi
Day12-Cisplatin
Day12-MMC
