# Supplementary figures and images for: Precision screening facilitates clinical classification of BRCA2-PALB2 binding variants with benign and pathogenic functional effects
Source: J Clin Invest. 2025 Apr 15;135(12):e181879. doi: 10.1172/JCI181879 (PMC12165785; doi:10.1172/JCI181879)

Full unedited blot/gel for Figure 4C

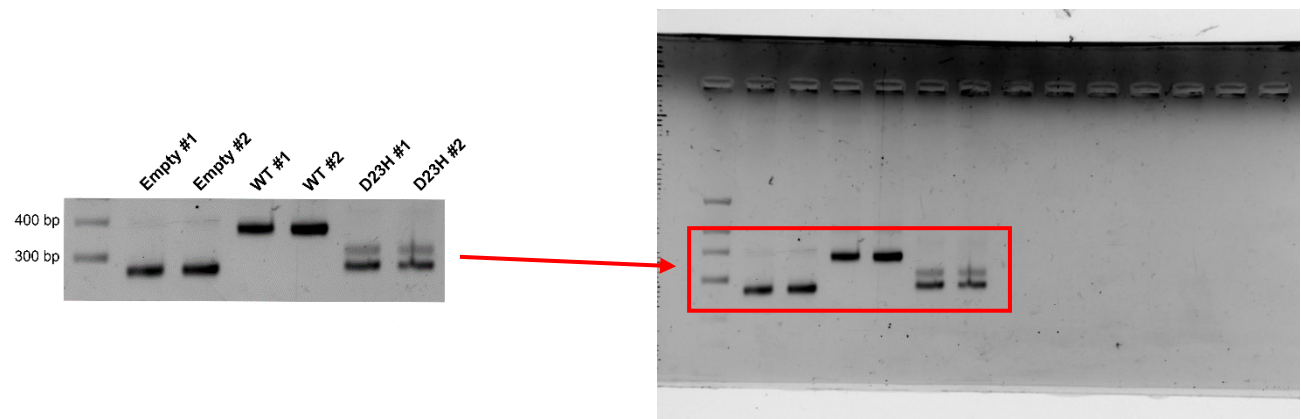

Full unedited blot/gel for Figure S1D

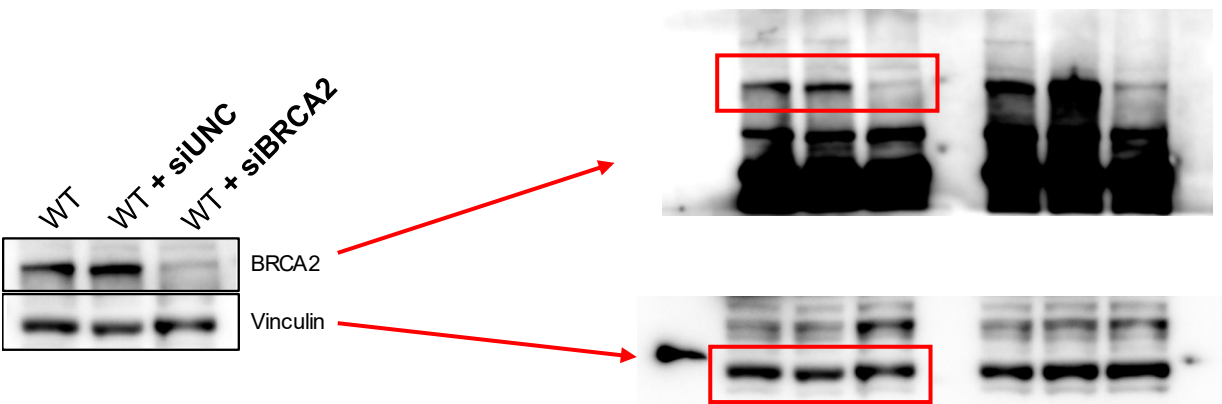

Supplement: Unedited blot and gel images [file jci-135-181879-s032.pdf]
